# Crystal structure via fluctuation scattering

**DOI:** 10.1107/S2052252524003932

**Published:** 2024-06-06

**Authors:** Patrick Adams, Tamar L. Greaves, Andrew V. Martin

**Affiliations:** a RMIT University, Melbourne, VIC 3000, Australia; Harima Institute, Japan

**Keywords:** serial crystallography, correlated fluctuations, molecular crystals, fluctuation X-ray scattering, iterative projection algorithms, X-ray cross-correlation analysis, structure-factor intensities, Bragg peak intensity

## Abstract

Crystallography is a quintessential method for determining the atomic structure of crystals. The most common implementation of crystallography uses single crystals that must be of sufficient size, typically tens of micrometres or larger, depending on the complexity of the crystal structure. The emergence of serial data-collection methods in crystallography, particularly for time-resolved experiments, opens up opportunities to develop new routes to structure determination for nanocrystals and ensembles of crystals. Fluctuation X-ray scattering is a correlation-based approach for single-particle imaging from ensembles of identical particles, but has yet to be applied to crystal structure determination. Here, an iterative algorithm is presented that recovers crystal structure-factor intensities from fluctuation X-ray scattering correlations. The capabilities of this algorithm are demonstrated by recovering the structure of three small-molecule crystals and a protein crystal from simulated fluctuation X-ray scattering correlations. This method could facilitate the recovery of structure-factor intensities from crystals in serial crystallography experiments and relax sample requirements for crystallography experiments.

## Abstract 2

Fluctuation X-ray scattering measures the correlation of scattered X-rays in diffraction experiments. Here, the Bragg peak intensity from fluctuation X-ray scattering correlations is recovered using an iterative algorithm.

## 1. Introduction

Understanding the atomic structure of molecules and materials is critical to many scientific fields, such as pharmacology, molecular biology, chemistry and materials science (Brink & Helliwell, 2019 ▸). Biomolecules, such as proteins, perform specific functions within the body. The atomic structure facilitates these functions and, hence, knowing the atomic structure of these biomolecules can inform how they interact. This forms the basis of structure-based drug design (Reynolds, 2014 ▸; Marrone *et al.*, 1997 ▸), where potential trial medicines are chosen based on targeting specific components of biomolecule structure. Through this process, compounds can be optimized to improve binding and specificity for the target component (Anderson, 2003 ▸).

X-ray crystallography is the dominant structure-determination technique for proteins. Of the over 212 000 deposited structures in the Protein Data Bank (PDB), over 180 000 were discovered with X-ray crystallography (Chapman & Fromme, 2017 ▸; Berman *et al.*, 2000 ▸). In this technique, a single crystal is rotated within an X-ray beam to obtain diffraction patterns of the crystal in all orientations. The diffraction patterns sample a slice of the reciprocal-space intensity function, which consists of a series of Bragg peaks. The intensity of the Bragg peaks is related to the electron density in the crystal (Warren, 1990 ▸), which can be used to construct a model of the atomic structure being investigated. To achieve 2–3 Å resolution, protein crystals need to be of the order of tens of micrometres in size (Holton & Frankel, 2010 ▸).

X-ray crystallography is limited by two interconnected factors: (i) X-ray damage to the crystals during data collection and (ii) the requirement for sufficiently large crystals (Chapman *et al.*, 2014 ▸). X-ray damage to the crystal can cause the loss of high-resolution Bragg peaks and induce structural changes in the atomic structure (Owen *et al.*, 2006 ▸). Some amino acids, the basic structural units of proteins, are more susceptible to X-ray damage than others (Weik *et al.*, 2000 ▸; Burmeister, 2000 ▸). This can affect the interpretation of the structure, particularly active sites in metallo-proteins (Yano *et al.*, 2005 ▸; Carugo & Carugo, 2005 ▸). Larger crystals are less susceptible to X-ray damage and scatter more strongly than smaller crystals (Holton, 2009 ▸). The scattered signal must be strong enough to overcome the noise of the background scattering. Increasing the signal-to-noise ratio can be accomplished by increasing the size of the crystals, or increasing the exposure time. However, increasing the exposure time necessarily increases the X-ray dose and, hence, potential damage to the crystal. Crystals have been cryogenically cooled to mitigate radiation damage as early as the 1960s in pre-synchrotron experiments (Low *et al.*, 1966 ▸). Improved X-ray sources at synchrotrons have made cryo-freezing critical in determining protein structure (Hendrickson, 2000 ▸). The structures of cryo-cooled crystals can be different from those at physiological temperatures and are not suitable for all time-resolved experiments (Botha *et al.*, 2015 ▸).

Serial crystallography is a development upon traditional crystallography, where the structure is determined by merging the diffraction patterns from many single crystals, rather than one crystal being rotated in the beam. The first serial crystallography experiments were conducted at ultra-fast ultra-bright X-ray sources called X-ray free electron lasers (XFELs). At these facilities, a solution of microcrystals is continuously streamed into an XFEL beam. When a femtosecond pulse of X-rays hits a single crystal in a random orientation, the exposure is fast enough to capture the diffraction of the crystal before it is destroyed in the beam (Chapman *et al.*, 2014 ▸). The diffraction patterns of many crystals in random orientations are collected individually, one crystal per exposure. Each crystal is in a different orientation, so each diffraction pattern measures a different slice through the reciprocal-space intensity function. By collecting thousands of diffraction shots of crystals in random orientations, the whole of the reciprocal-space intensity function can be sampled (Schriber *et al.*, 2022 ▸). There are a variety of sample-delivery methods for serial femtosecond crystallography experiments. These include liquid or gas injection (Nogly *et al.*, 2016 ▸; Vakili *et al.*, 2020 ▸), and fixed target systems such as tape drives (Beyerlein *et al.*, 2017*a* ▸) and membrane targets (Roedig *et al.*, 2017 ▸; Fuller *et al.*, 2017 ▸).

There are several advantages of serial femtosecond crystallography over traditional X-ray crystallography with a single crystal. XFELs are of the order of billions of times brighter than typical synchrotron sources (Boutet *et al.*, 2018 ▸) and can measure smaller crystals than can be achieved at synchrotron sources (Spence, 2017 ▸). Smaller crystals also facilitate chemical mixing experiments (Stagno *et al.*, 2017 ▸), where diffusion of a ligand into a crystal before injection can induce conformational change in the investigated structure. Radiation damage effects are mitigated by capturing the diffraction before destruction, which facilitates room-temperature experiments (Chapman *et al.*, 2014 ▸). Room-temperature crystallography allows for time-resolved studies of protein and enzymatic function (Kern *et al.*, 2012 ▸), providing greater insight into structural properties of biomolecules. The development of serial femtosecond crystallography has led to serial crystallographic methods being applied at synchrotron sources (Botha *et al.*, 2015 ▸). Although radiation damage cannot be outrun as it is in XFEL experiments, measuring an ensemble of crystals can reduce the radiation dose on a per crystal basis (Weinert *et al.*, 2017 ▸).

A potential problem with crystallography methods comes with multi-crystal diffraction shots. Crystal diffraction patterns need to be indexed, which is a process that determines the location of each Bragg peak from a crystal in 3D reciprocal space. If more than one crystal is diffracting in the beam, diffraction patterns could be misindexed and reduce the quality of the recovered structure (Nam, 2022 ▸). This can occur in both serial and traditional crystallography experiments. Frequently, crystals grow in clusters or have some degree of mosaicity. Indexing algorithms such as *XGANDALF* (Gevorkov *et al.*, 2019 ▸) and *FELIX* (Beyerlein *et al.*, 2017*b* ▸) can index multi-crystal diffraction patterns. However, experimental demonstrations of these algorithms typically handle ten or less crystals per diffraction shot (Nam, 2022 ▸; Beyerlein *et al.*, 2017*b* ▸).

Powder diffraction is another method of structure determination that measures ensembles of crystals. In a powder diffraction experiment, a powder of microcrystals is exposed to an X-ray source. The diffraction from each crystallite is measured simultaneously, which causes diffraction in the form of isotropic Debye–Scherrer rings (Warren, 1990 ▸). The integrated intensity around each ring as a function of scattering angle is calculated. Rietveld refinement (Rietveld, 1969 ▸) is then used to determine the crystal structure within the powder. Powder diffraction is typically used for small unit-cell crystals, such as organic chemical crystals or minerals. Peak overlap in the integrated intensity can occur if the unit cell is too large (Keen, 2020 ▸). Biomolecules, such as proteins, have large unit cells compared with small chemical crystals. As such, there have only been 19 protein structures solved via powder diffraction (Spiliopoulou *et al.*, 2020 ▸).

Between crystallography and powder diffraction methods, the number of crystals within the beam is critical in opposing ways. Modern serial crystallography experiments are hindered if there are too many crystals diffracting at once, while still requiring the diffraction from many crystals individually in many orientations. Conversely, in powder diffraction methods there is a minimum number of crystals required to form isotropic diffraction rings. Incomplete or ‘spotty’ rings can cause miscalculation of the integrated intensity, which can lead to poor structure recovery (Evans & Evans, 2004 ▸).

Fluctuation X-ray scattering (FXS) is a diffraction analysis technique that could potentially overcome the issue of measuring too few or too many crystals. FXS was originally devised to recover the structures of single particles in solution (Kam, 1977 ▸) and is often used in conjunction with ensemble measurements. This is achieved by calculating the angular intensity correlation functions of ensembles of particles, averaged over many patterns (Zaluzhnyy *et al.*, 2019 ▸). FXS has been used to study the structures of a variety of materials, such as local structures within carbon amorphous materials (Martin *et al.*, 2020*a* ▸), self-assembled lipid phases (Martin *et al.*, 2020*b* ▸), gas-injected single particles (Starodub *et al.*, 2012 ▸) and viruses (Seibert *et al.*, 2011 ▸). FXS provides many advantages to other scattering techniques, as it allows for many particles to be observed within a single exposure, relaxing constraints on the number of particles in the beam.

In this work, we developed an iterative algorithm that extracts the Bragg peak intensity from FXS correlation functions, based on an approach developed by Donatelli *et al.* for single particles (Donatelli *et al.*, 2015 ▸, 2017 ▸). Our algorithm relies on the known location of Bragg peaks in reciprocal space, which can be determined from known unit-cell parameters. We calculated correlation functions from the Bragg intensities of previously known small-molecule crystal structures. The correlation functions were used as input to the iterative algorithm, which recovered the Bragg peak intensities. We then used established methods of structure refinement on the recovered intensities to compare with the original known structures. Our algorithm could potentially be used to obtain crystal structures from powder-like samples that do not meet the requirements for standard crystallography, due to insufficiently sized crystals or the number of crystals in the beam. This is a step towards crystallographic structure determination from multi-crystal patterns that avoids multi-crystal indexing.

## 2. Theory and methods

### 2.1. Fluctuation X-ray scattering

FXS is an X-ray scattering technique that measures the correlation between the intensities of pairs of points within a diffraction pattern with respect to the scattering-length magnitudes *q*_1_ and *q*_2_ and the angle between the scattering vectors ψ (Zaluzhnyy *et al.*, 2019 ▸). In a typical FXS experiment, many identical structures in different orientations are measured in each diffraction pattern. FXS analysis methods often assume that the orientation distribution of the structures is uniform and random. The correlation function *C*(*q*_1_, *q*_2_, ψ) is then given by 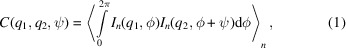
where 〈〉_*n*_ represents the average over *n* diffraction patterns, denoted by *I*(*q*, ϕ) in polar coordinates (Kirian, 2012 ▸). If each individual diffraction image contains multiple dilute particles per exposure with a uniform orientation distribution, then the multiple-particle correlation function converges to the single-particle correlation function after averaging (Kam, 1977 ▸).

**Figure 1:**
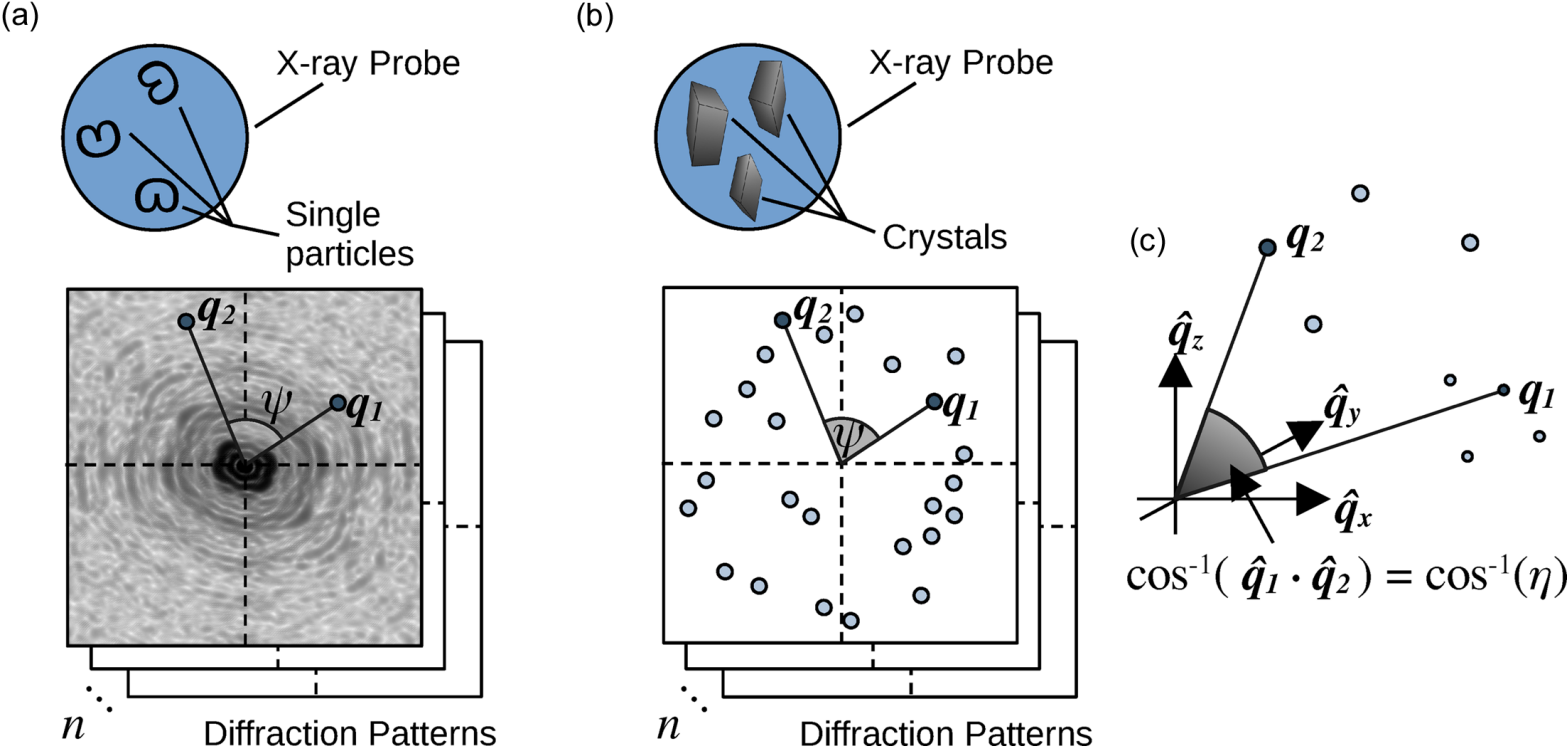
(*a*) Correlation calculated from single structures that have continuous reciprocal-space intensity on a 2D detector, as has been previously used for single particles or amorphous materials. The angular correlation is calculated with respect to scattering magnitudes *q*_1_ and *q*_2_, and angle ψ, according to equation (1). (*b*) Correlation of Bragg peaks on a 2D detector. The intensities of every pair of points are multiplied and summed together according to their scattering magnitudes *q*_1_ and *q*_2_, and the angle ψ between them, according to equation (2). This illustration would apply to FXS-based crystallography experiments. (*c*) Correlation of Bragg peaks in 3D reciprocal space. Intensities are similarly multiplied and summed together according to equation (4). This method was used in this article for algorithm development because it is efficient to compute and it provides the ‘ground truth’ of correlation intensity.

FXS has previously been used to study the diffraction of single particles (Kurta *et al.*, 2017 ▸) and amorphous materials (Wochner *et al.*, 2009 ▸). The reciprocal-space intensity function of these scatterers is continuous, as illustrated in Fig. 1 ▸(*a*), and so a continuous integral about ϕ is used in equation (1). However, in a crystallography experiment, a process of peak finding is conducted that produces a list *Q*_2*D*_ of peaks **q**_*i*_ = (*q*_*x*_, *q*_*y*_) ∈ *Q*_2*D*_ within a single diffraction pattern. The scattering magnitude *q*_*i*_ = |**q**_*i*_| of each peak can be calculated using the sample-to-detector distance and X-ray wavelength, and each peak has an integrated intensity *I*(**q**_*i*_). In this case, it is convenient to define the correlation function *C*(*q*_1_, *q*_2_, ψ) in terms of a double sum over all pairs of peaks in the peak list averaged over *n* diffraction patterns, 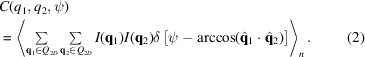
The Dirac delta function δ acts as a sifting function that only includes the correlations between **q**_1_ and **q**_2_ if the angle between the vectors is equal to ψ, as illustrated in Fig. 1 ▸(*b*). This essentially replaces the continuous integral with a discrete sum over the peaks observed within a single 2D diffraction pattern, averaged over many diffraction patterns.

We can equivalently calculate the correlation function from a list of 3D Bragg peaks, as described by Adams *et al.* (2020 ▸). Let *Q*_*hkl*_ be a list of reciprocal vectors **q**_*hkl*_ = (*q*_*x*_, *q*_*y*_, *q*_*z*_) ∈ *Q*_*hkl*_, where *h*, *k* and *l* are the Miller indices for the Bragg peaks. We will denote *Q*_*hkl*_(*q*) as a subset of *Q*_*hkl*_ that has vectors with magnitude *q*: 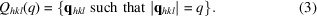
Then the correlation function *C*(*q*_1_, *q*_2_, η) is given by 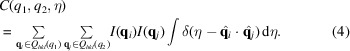
Similar to equation (2), this correlation-function calculation is a double sum over all pairs of peaks in the peak list *Q*_*hkl*_, as demonstrated in Fig. 1 ▸(*c*). In equations (1) and (2) the angular coordinate ψ is in units of radians, but in equation (4) the coordinate η is a dimensionless quantity between −1 and 1, or 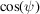. The re-parametrization of the angular coordinate will become useful when describing the correlation function in terms of Legendre polynomials in equation (8). There are some factors to consider when establishing the equivalence of the 2D and 3D correlation functions. Firstly, equation (4) is related to the 2D function in equation (2) by a multiplicative factor of |**q**_1_||**q**_2_|, which accounts for the curvature of the Ewald sphere. Secondly, if the angular coordinate of the 3D correlation function is sampled over ψ, it is related to the 2D correlation function by a multiplicative factor of 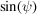. The 3D correlation function presents an ideal or ‘ground truth’ correlation function and is the convergence point of the 2D correlation function over many diffraction patterns. For the purposes of developing and testing our algorithm, we will be using the 3D correlation function here.

### 2.2. Spherical harmonics

The 3D reciprocal-space intensity function *I*(*q*, θ, ϕ) denotes the diffracted intensity from an object, and can be expanded in terms of spherical harmonic functions *Y*_*lm*_(θ, ϕ) and spherical harmonic coefficients *I*_*lm*_(*q*) (Sloan, 2013 ▸). The decomposition is given by 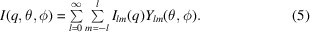
The spherical harmonic functions are an orthogonal set of real basis functions determined by
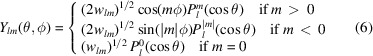
where 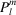 are the associated Legendre polynomials and *w*_*lm*_ are normalization constants given by 
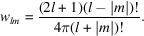
The spherical harmonic coefficients are identified by 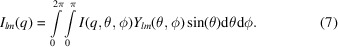
We will denote the forward and backward spherical harmonic transformations as 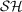 and 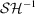, respectively. That is 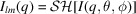
and 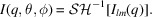
We can define the correlation function *C*(*q*_1_, *q*_2_, η) of an intensity function in terms of the spherical harmonic coefficients *I*_*lm*_(*q*). This derivation is described in the literature by Saldin *et al.* (2009 ▸) ▸, and produces an expression for the harmonic order matrix *B*(*q*_1_, *q*_2_, *l*), given by
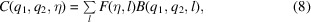
where 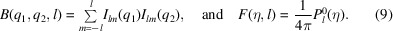
The relationship between the 2D diffraction patterns in polar coordinates *I*(*q*, ϕ) can be expressed in terms of the 3D reciprocal-space function of the molecule on the Ewald sphere *I*[*q*, θ(*q*), ϕ] through a re-parametrization of the θ coordinate as a function of *q* and an arbitrary wavenumber *k*, stated by 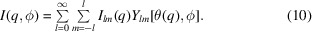
The θ(*q*) re-parametrization is determined by 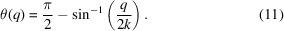
An explicit mathematical description of how the 2D scattering correlation function is related to the Ewald sphere is described by Saldin *et al.* (2009 ▸).

### 2.3. Computation specifics

We represent the correlation function *C*(*q*_1_, *q*_2_, η) as a 3D matrix array with two radial coordinates, *q*_1_ and *q*_2_, and a cosine coordinate, η. The size of this matrix is defined by the integer parameters *n*_*q*_ and *n*_η_, which determine the number of radial and angular sampling points of the correlation function. The parameter 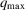 sets the maximum *q* value for the correlation function and is directly proportional to the minimum resolution *d* of the electron density, 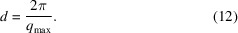
Hence, high-resolution features within the structure are related to Bragg peaks with large scattering magnitudes.

The reciprocal-space intensity function is also represented as a 3D matrix array, with a radial coordinate *q* and two angular coordinates, θ and ϕ. The size and sampling of the radial axis is defined by *n*_*q*_ and 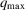, similar to the correlation function. The azimuthal angular coordinate ϕ is sampled over *n*_ϕ_ points between 0 and 2π, and the longitudinal angular coordinate θ is sampled over *n*_θ_ points between 0 and π. For consistency with the Driscoll–Healy spherical grid format (Driscoll & Healy, 1994 ▸), we require that *n*_ϕ_ = 2*n*_θ_. Using this format, the spherical harmonic transformations are invertible up to a spherical harmonic limit *n*_*l*_ that is half of *n*_θ_. That is, *n*_ϕ_ = 4*n*_*l*_.

Rearranging equation (8) to solve for the harmonic order matrix, we invert the *F* matrix and solve the following equation: 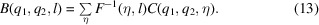
We calculate the Moore–Penrose pseudo-inverse of a *F* matrix (Ben-Israel & Greville, 2003 ▸) because *F* is a non-square matrix.

### 2.4. Iterative projection algorithms

To recover the reciprocal-space intensity function *I*(*q*, θ, ϕ) of a crystal given the scattering correlation function *C*(*q*_1_, *q*_2_, η), we will use an iterative projection algorithm. Iterative projection algorithms solve optimization problems that can be represented as the intersection between sets. For each set, a projection operator is defined that maps any given element to the closest element in the set. An algorithm can be formulated by applying the projection operators in different combinations to iteratively search for the intersection between the sets. The projection operators are typically formed from known properties of the solution, or constraints. See Marchesini (2007 ▸) for a detailed overview and evaluation of iterative projection algorithms.

**Figure 2:**
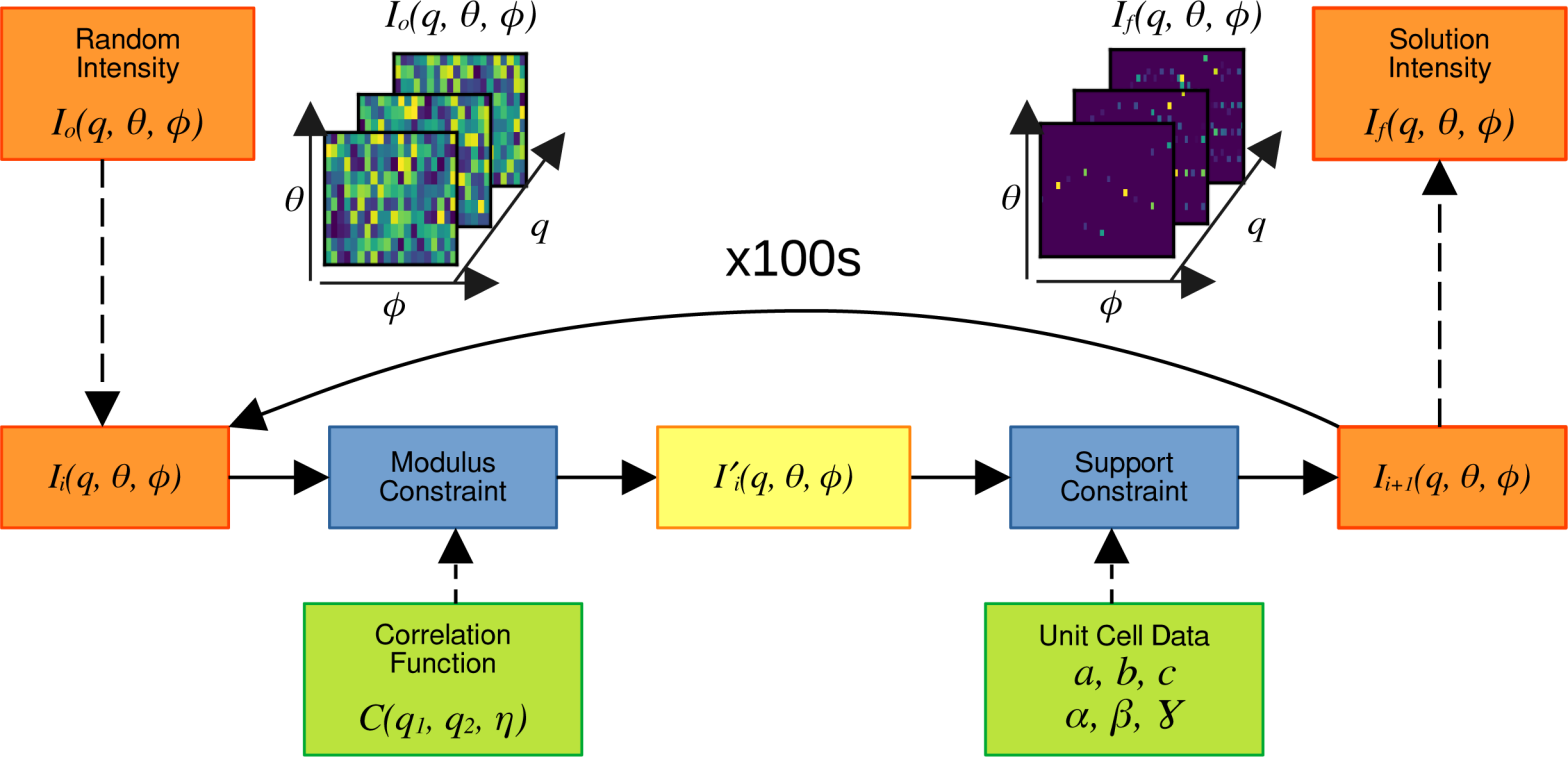
An overview of the iterative algorithm used in this work. A modulus constraint and a support constraint, shown in the blue boxes, are constructed from the scattering correlation function *C*(*q*_1_, *q*_2_, η) and unit-cell parameters *a*, *b*, *c*, α, β, γ, respectively, shown in the green boxes. Together, they are used in an iterative algorithm to recover the reciprocal-space intensity *I*_*f*_(*q*, θ, ϕ) of a single crystal from a random initial intensity *I*_*o*_(*q*, θ, ϕ), shown in the orange boxes. The yellow box indicates an intermediate intensity function that is obtained after applying the modulus constraint but before the support constraint.

Iterative algorithms have also previously been used in conjunction with scattering correlation analysis to reconstruct the electron density of single particles (Donatelli *et al.*, 2015 ▸, 2017 ▸). Our algorithm is designed to recover the reciprocal-space intensity function of a crystal, using the spherical harmonic relationship between the scattering correlation function and the intensity function, and the sparse support constraint of known Bragg peak locations. An overview of the algorithm is presented in Fig. 2 ▸.

#### 2.4.1. Modulus constraint

**Figure 3:**
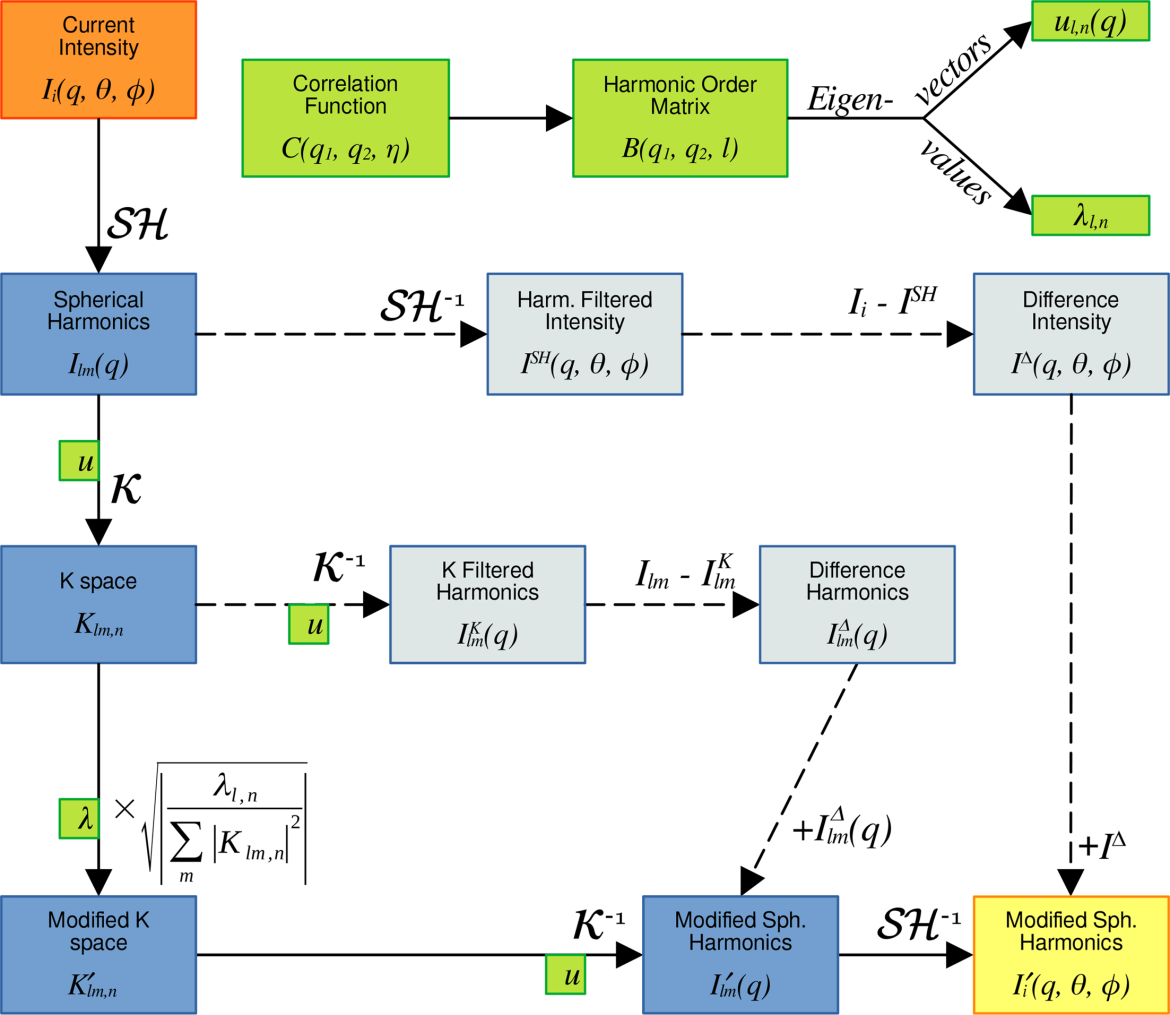
A flowchart illustrating the steps of the modulus constraint. The process begins with the current intensity function shown in the orange box and follows the solid black arrows between the dark blue boxes. Dashed arrows between the light blue boxes indicate additional steps required to account for lossy transformation processes. The green boxes illustrate the calculation of eigenvectors and eigenvalues from the harmonic order matrix and the correlation function, and where they are used in the transformation steps. After applying the modulus constraint, the intermediate intensity function shown in the yellow box is produced.

The modulus-constraint projection operator *P*_*m*_ modifies an intensity function *I*_*i*_(*q*, θ, ϕ) so that the spherical harmonic coefficients *I*_*lm*_(*q*) of the intensity function are consistent with the harmonic order matrices *B*(*q*_1_, *q*_2_, *l*). This process is illustrated in Fig. 3 ▸, following the solid arrows between the blue boxes.

The application of *P*_*m*_ begins by first decomposing the current intensity function *I*_*i*_(*q*, θ, ϕ) into a set of spherical harmonic coefficients *I*_*lm*_(*q*), given by 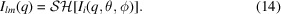
For each degree *l*, the *q*_1_, *q*_2_ indices of the harmonic order matrices are used for the rows and columns of a 2D matrix, respectively. This 2D matrix is decomposed into eigenvectors and eigenvalues as a function *q* with respect to one of the *q* indices. The choice of which *q* index is irrelevant is due to symmetry through *q*_1_ = *q*_2_. These eigenvectors are denoted *u*_*l*,*n*_(*q*), and the associated eigenvalues are denoted λ_*l*,*n*_. Next, the eigenvectors are used as a set of basis vectors to expand the spherical harmonic coefficients into a set of new coefficients *K*_*lm*,*n*_. This basis transformation is denoted by κ and shown by 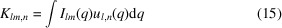
and 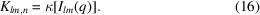
The inverse basis expansion κ^−1^ is determined by 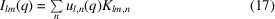
and 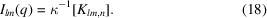
Once the *K*_*lm*,*n*_ coefficients have been calculated, they are scaled by the eigenvalues λ_*l*,*n*_ to make a new set of modified *K*′_*lm*,*n*_ coefficients, 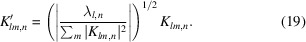
The modified *K*′_*lm*,*n*_ coefficients are converted back to modified spherical harmonic coefficients *I*′_*lm*_(*q*) by 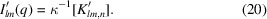
The spherical harmonic coefficients *I*′_*lm*_(*q*) are now consistent with spherical harmonic coefficients in the harmonic order matrix *B*(*q*_1_, *q*_2_, *l*). Finally, the spherical harmonic coefficients *I*′_*lm*_(*q*) are used to obtain an updated intensity function *I*′_*i*_(*q*, θ, ϕ):
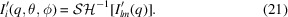


#### 2.4.2. Lossy basis expansions

For each basis-expansion step, there are a finite number of terms that can be calculated. For example, the number of eigenvalues and eigenvectors that are calculated in the κ expansion depends on the number of radial-sampling points *n*_*q*_ that sample the intensity and correlation functions. The maximum number of spherical harmonic coefficients *n*_*l*_ that can be calculated is limited by the number of angular-sampling points *n*_θ_, *n*_ϕ_ in the intensity function. In both of these basis expansions, higher-order terms are not accounted for and not constrained by the modulus constraint. To account for the higher-order terms, there is a series of extra steps that must be completed, which are illustrated in Fig. 3 ▸ by the dashed arrows.

After completing the first spherical harmonic decomposition to calculate *I*_*lm*_(*q*) up to *n*_*l*_ harmonic coefficients, the reciprocal-space intensity is recomposed from the coefficients to produce a low-pass filtered intensity function 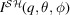, given by 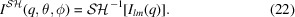
The difference intensity function *I*^Δ^(*q*, θ, ϕ) is calculated by subtracting the low-pass filtered function from the starting function *I*_*i*_(*q*, θ, ϕ), 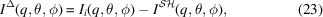
so that *I*^Δ^(*q*, θ, ϕ) contains the contributions of higher-order harmonic terms. These higher-order harmonic terms are then added to the next iteration of the intensity function, 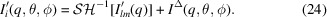
Through the κ basis expansion, there are a limited number of eigenvectors used as basis vectors for the expansion. After expanding to the *K*_*lm*,*n*_ coefficients, the spherical harmonic coefficients filtered by the expansion 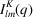 are calculated: 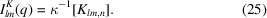
The difference terms 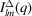 are calculated by subtracting the κ filtered terms from the original spherical harmonic coefficients before the κ expansion, 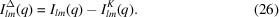
The difference terms 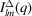 are then added to the harmonic coefficients after scaling by the eigenvalues, 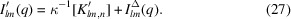


#### 2.4.3. Support constraint

The support projection operator *P*_*s*_ modifies the intensity function *I*(*q*, θ, ϕ) to retain the intensity within a small volume around each Bragg peak and sets the intensity to 0 everywhere else. A volume *V*_*hkl*_ is centred on the Bragg peak **q**_*hkl*_ = (*q*_*hkl*_, θ_*hkl*_, ϕ_*hkl*_) and extends in each spherical coordinate axis by a small amount (*q*_*V*_, θ_*V*_ and ϕ_*V*_). The volume *V*_*hkl*_ is provided by
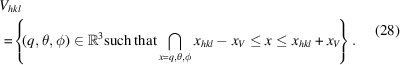
Let *M* be a binary support mask that includes all the volumes *V*_*hkl*_ around each Bragg peak **q**_*hkl*_, given by 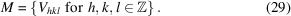
The support constraint can be applied to the intensity function *I*(*q*, θ, ϕ) with the following equation:
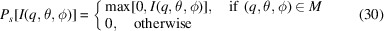
Within the support constraint, we also apply a global positivity constraint using the max function, such that any intensity values that are negative are set to 0.

#### 2.4.4. Iterative schemes

After constructing our projection operators *P*_*m*_ and *P*_*s*_, the next step is to apply these constraints within an iterative scheme, such as the error reduction (ER) or hybrid input–output (HIO) algorithms (Marchesini, 2007 ▸). ER is the simpler of the two iterative schemes, where the projection operators are sequentially applied on the intensity function *I*(*q*, θ, ϕ), as described by 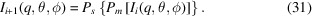
It is known that ER converges to the closest minima, and only converges to the global solution if it starts near the solution.

The HIO algorithm is based on nonlinear feedback theory and does not stagnate at local minima (Marchesini, 2007 ▸). HIO is given by
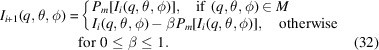
We assume a β value of 0.9 for all uses of HIO presented here, which has been found to be successful in previous phase-retrieval studies (Chen *et al.*, 2007 ▸). Frequently, iterative algorithms are run with alternating schemes and can be described with an iterative algorithm recipe, *e.g.* 20 iterations of the HIO scheme, followed by two iterations of the ER scheme, repeated five times.

### 2.5. Target structures

To test the algorithm, we used the structures of three chemical crystals from the Crystallography Open Database (Gražulis *et al.*, 2009 ▸). These structures were silver nitrate with a ligand, aluminophosphate and a dipeptide precursor. We selected structures with different cell sizes, lattice types, symmetries and constituent atoms, as outlined in Table 1 ▸. We calculated the structure factors *F*_*hkl*_ for each crystal structure using *VESTA* (Momma & Izumi, 2008 ▸), to a *d* resolution of 0.3 Å for the silver nitrate structure and 0.5 Å for the aluminophosphate and dipeptide precursor structures. This corresponds to a 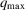 of 22 Å^−1^ for the silver nitrate structure and 12.6 Å^−1^ for the aluminophosphate and dipeptide precursor structures. Due to the different cell sizes, each structure had a different number of scattering vectors with scattering magnitude 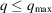. The silver nitrate had 121 382 vectors, the aluminophosphate had 44 586 vectors and the dipeptide precursor had 89 618 scattering vectors.

### 2.6. Correlation calculation and algorithm parameters

The algorithm and correlation calculation scripts are available in the open-source Python package *SCORPY* (Adams, 2022 ▸). All demonstrations of the algorithm were run on an HP Pavilion 15 laptop, with 16 GB of RAM and an eighth-generation Intel Core i7 processor.

We calculated the correlation functions from the Bragg peak intensities according to equation (4), using the structure factors generated in *VESTA*.

For each correlation function, the *n*_*q*_ and *n*_η_ parameters were 300 and 5760 sampling points, respectively. The correlation-function calculation for the silver nitrate, aluminophosphate and dipeptide precursor samples took ∼34, 6 and 25 h, respectively. After calculating the correlation functions, we computed the harmonic order matrices *B*(*q*_1_, *q*_2_, *l*) for each sample. These matrices were calculated up to *l* = 250 spherical harmonics to satisfy the Driscoll–Healy grid format. The magnitude of the harmonic order matrices for *l* ≥ 45 was small relative to those with *l* < 45. The reconstructions improved when the matrices for *l* ≥ 45 were set to 0. The eigenvectors *u*_*l*,*n*_(*q*) and eigenvalues λ_*l*,*n*_ used within the modulus constraint were calculated from these harmonic order matrices.

To run the algorithm, we initialized a random intensity function with *n*_*q*_ = 300, *n*_θ_ = 500 and *n*_ϕ_ = 1000. The random intensity values ranged between −1 and 1. A support mask *M* was created from the unit-cell parameters for each sample that included all peaks with 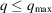. For each peak, the support mask included a cubic volume that was 5 voxels wide and centred on the peak location. A single algorithm run consisted of 120 iterations of HIO, which took ∼13 h. Eight runs were performed for each of the three samples with different random initial intensities per run.

### 2.7. Structure refinement

The crystal structure *R* factor compares the structure-factor intensities from a model structure *I*_calc_ to the intensities observed in an experiment *I*_obs_ (IUCr, 2017 ▸). It is given by 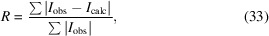
where the sums are calculated over all the Bragg peaks. The *R* factor was calculated at every iteration of the algorithm and is quoted as a measure of model quality. Here we use the same *R* factor, substituting the target intensities for *I*_calc_ and the intensities recovered by the algorithm for *I*_obs_. Typical values for *R* factors change depending on the structure being refined. For protein model refinement, an *R* factor of ∼0.2 is considered a desirable target for 2.5 Å resolution. Small organic molecule crystals frequently refine to an *R* factor of less then 0.05 (IUCr, 2017 ▸).

To compare solutions generated from independent runs of the algorithm, we will use an *R*_iso_ factor. A low value of *R*_iso_ indicates convergence of intensities to a uniform solution. The expression for the *R*_iso,*ij*_ factor between independent solutions *i* and *j* is determined by 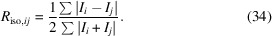
We used *SHELXL* (Sheldrick, 2008 ▸) for structural refinement from the crystal intensities that were recovered from the algorithm. The average atomic displacement was calculated from the difference between the atomic locations in the final recovered structure from *SHELX* and the target structure. Let *T*_*i*_ ∈ *T* denote the (*x*, *y*, *z*) coordinates of the *i*th atom in the target structure *T* with *N* total atoms. Similarly, denote the atoms in the recovered structure by *P*_*i*_. Then the mean atomic displacement is given by 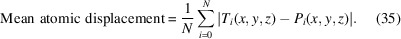


## 3. Results

### 3.1. Recovered intensities

**Figure 4:**
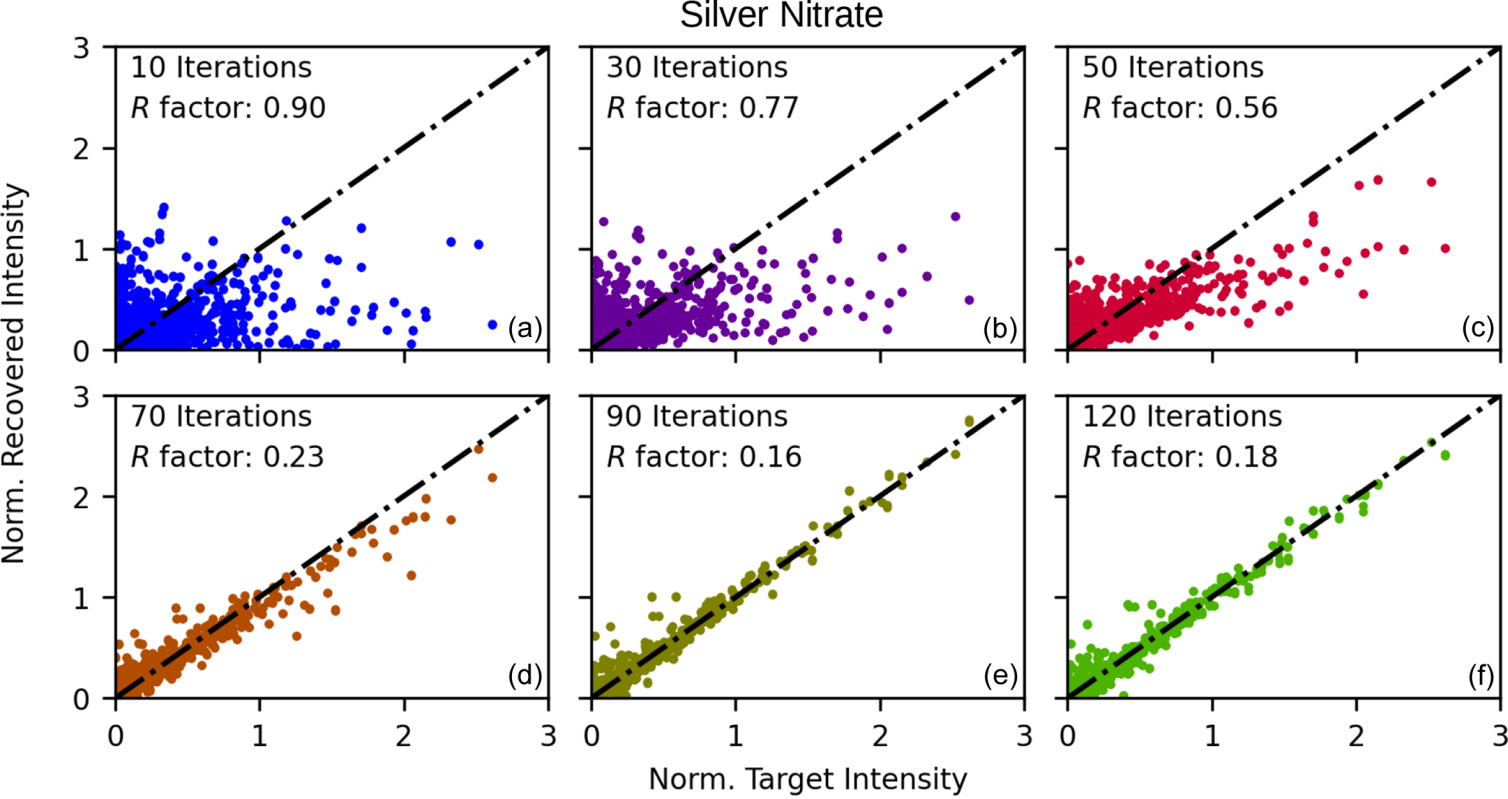
The recovered Bragg intensities versus the target intensities for the silver nitrate structure at different numbers of iterations. Each plot is generated from the intensities after different numbers of algorithm iterations, illustrating that as the algorithm progresses, the recovered intensities approach the *y* = *x* line and, hence, approach the target intensities. Axis units are arbitrary.

The plots in Fig. 4 ▸ show the results of the recovered Bragg intensities for the silver nitrate sample at different iterations during the algorithm. For each Bragg peak intensity, the intensity values of each run were averaged and plotted against the target intensity values. Initially, after ten iterations, the intensities are poorly recovered. This is illustrated in Fig. 4 ▸(*a*). However, after further iterations, we observe that the intensities approach the *y* = *x* line, indicating that each Bragg peak intensity is approaching its associated target intensity. This is illustrated in Figs. 4 ▸(*b*)–4 ▸(*f*). Similar convergence behaviour was observed for the aluminophosphate and dipeptide precursor intensities.

**Figure 5:**
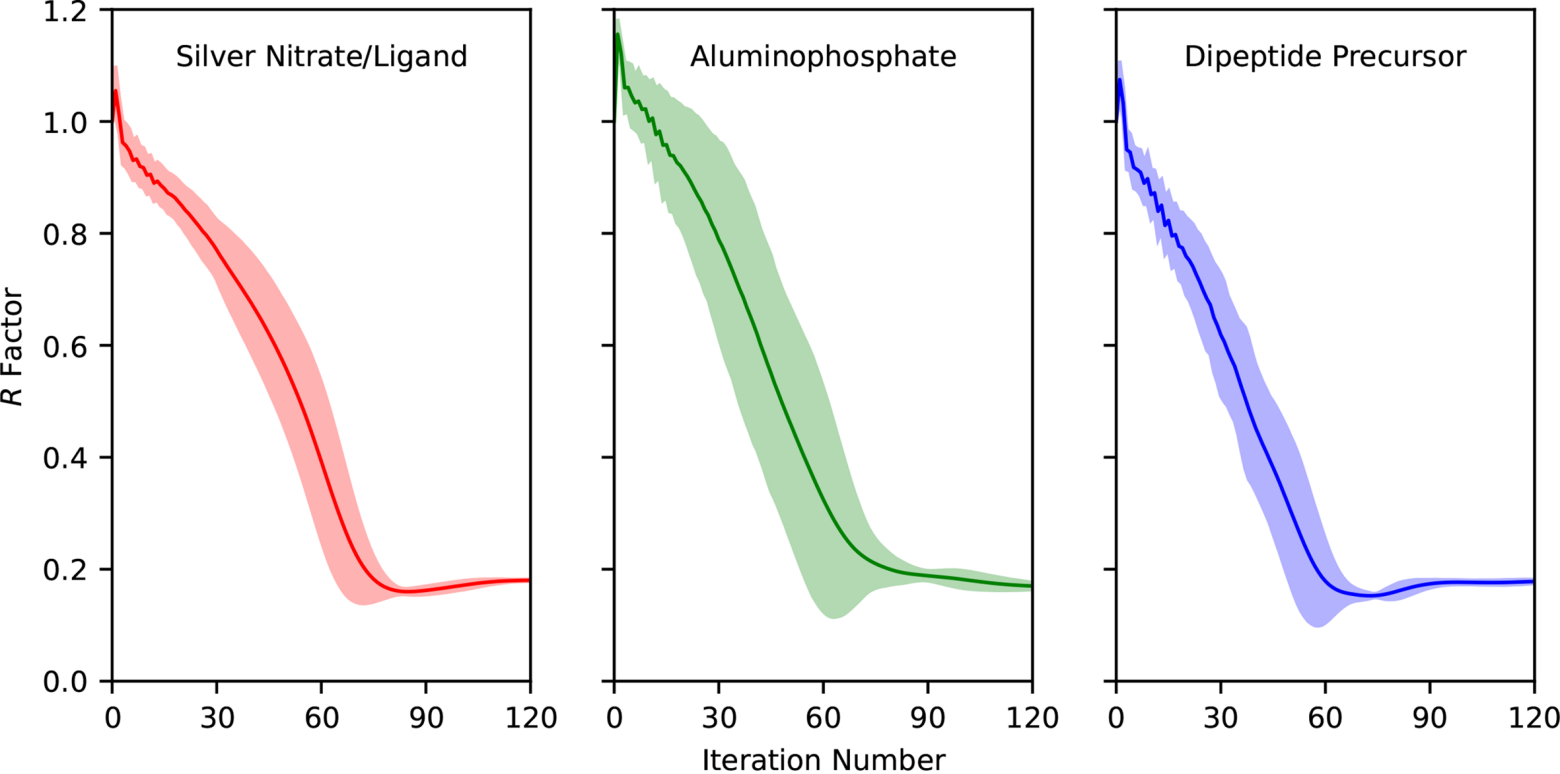
Plots of the *R* factors versus iteration number for the silver nitrate, aluminophosphate and dipeptide precursor reconstructions, left to right, respectively. The solid line traces the average *R* factor over eight independent reconstructions with the same parameters but different random starts. The shaded regions above and below the average indicate ±3 standard deviations from the mean of the eight runs. The *R* factor calculated at each step decreases, indicating that the reconstructed intensities are approaching the target intensities.

The *R* factor at every iteration was calculated for all of the independent runs, comparing the recovered intensities *I*_obs_ to the target intensities *I*_calc_, as in equation (33). Fig. 5 ▸ shows the average *R* factor over the course of the algorithm. The shaded regions indicate ±3 standard deviations from the average, estimated from the independent runs. Overall, the *R* factor decreases as the algorithm progresses. Each sample exhibits a minimum *R* factor of ∼0.2 between 60 and 90 iterations. After this, the *R* factor either continues to marginally increase, as in the silver nitrate and dipeptide precursor samples, or continues to marginally decrease, as in the aluminophosphate sample. An interesting feature within the plots in Fig. 5 ▸ is that the standard deviation error remains small after the minimum *R* factor is reached, indicating that all eight independent runs are close to the same intensity solution. This occurs at ∼85 iterations for the silver nitrate sample, 90 iterations for the aluminophosphate sample and 70 iterations for the dipeptide precursor.

The average *R*_iso_ factor was calculated between the final intensities of every pair of independent runs for each sample. The intensity solutions from the silver nitrate, aluminophosphate and dipeptide precursor runs had an average *R*_iso_ of 0.01 ± 0.002, 0.02 ± 0.004 and 0.02 ± 0.003, respectively.

### 3.2. Recovered structures

**Figure 6:**
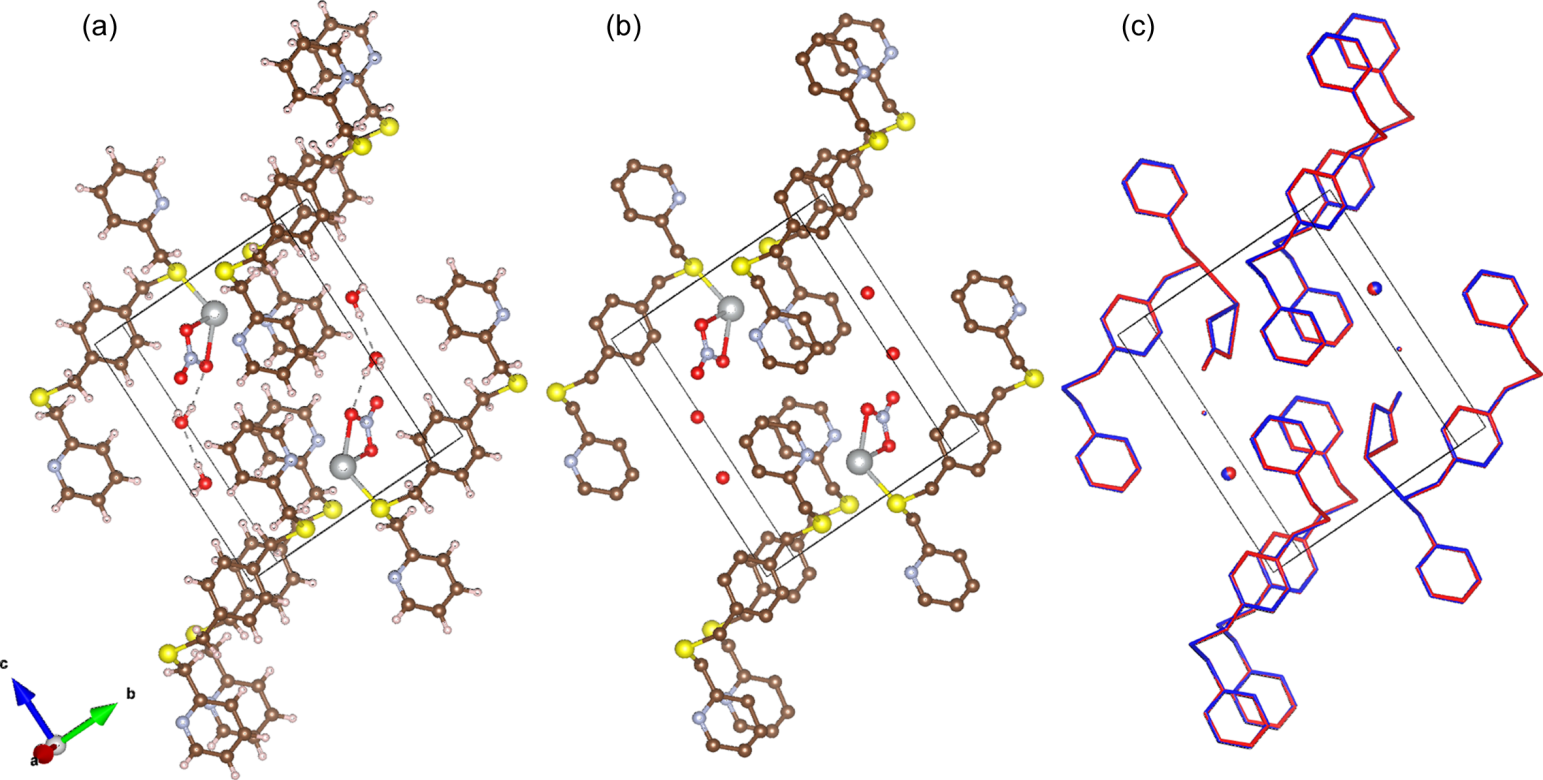
(*a*) The target and (*b*) the recovered structures for the silver nitrate sample. (*c*) The overlay of the blue target and red recovered structures.

**Figure 7:**
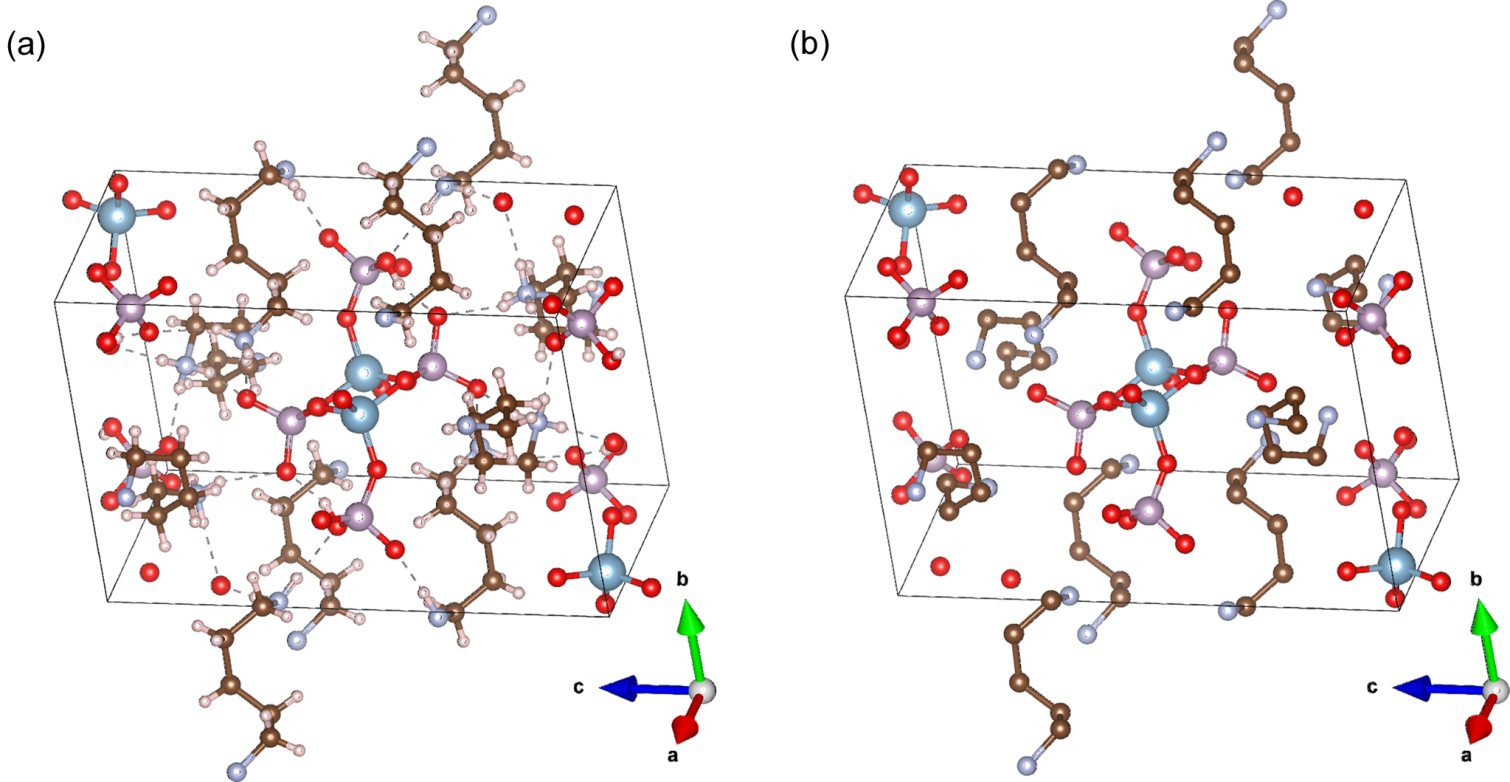
(*a*) The target and (*b*) the recovered structures for the aluminophosphate sample.

The target structure for the silver nitrate sample is shown in Fig. 6 ▸(*a*). Compared with the structure generated from the algorithm intensities [Fig. 6 ▸(*b*)], the figure shows that the structure was successfully recovered. This is further illustrated in the overlay of the structures in Fig. 6 ▸(*c*), with the blue target structure matching the red recovered structure quite closely. The structures for aluminophosphate and the dipeptide precursor were similarly successful, as illustrated in Figs. 7 ▸, 8 ▸ and 9 ▸. This demonstrates that the algorithm can recover samples with different unit-cell symmetries.

**Figure 10:**
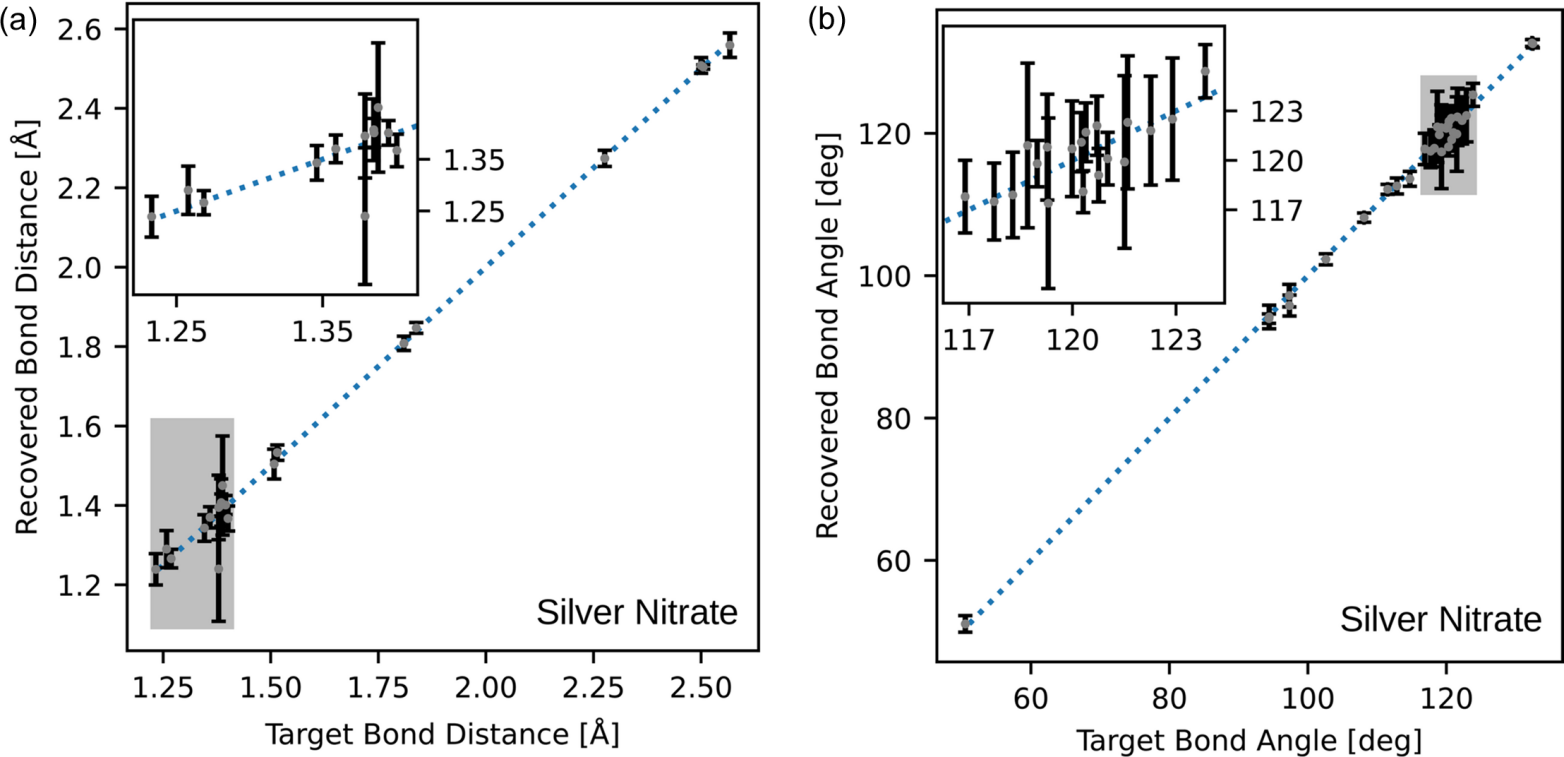
Comparison of (*a*) bond lengths and (*b*) bond angles of the target and the average structure for the silver nitrate sample. Error bars are ±3 standard deviations, estimated from the eight reconstructions. The insets show a zoomed vision of the grey areas in the respective plots.

To quantify the accuracy of the recovered structures, the recovered bond distances and angles have been plotted against the target values for the silver nitrate, aluminophosphate and dipeptide precursor samples in Figs. 10 ▸, 11 ▸ and 12 ▸, respectively. Across these figures, it is evident that some bond lengths and angles are accurately reconstructed, while others have larger standard deviations.

**Figure 11:**
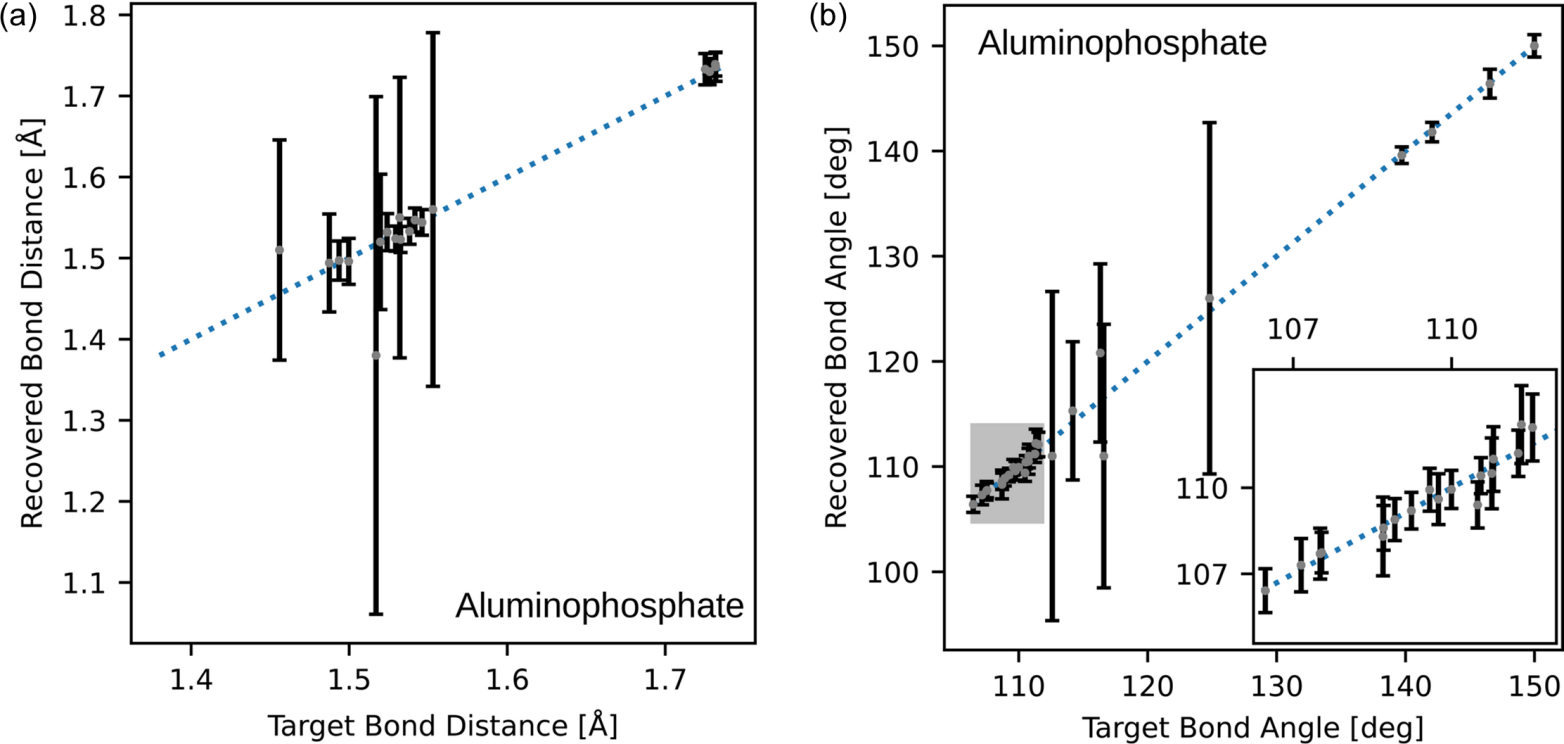
Comparison of (*a*) bond lengths and (*b*) bond angles of the target and the average structure for the aluminophosphate sample. Error bars are ±3 standard deviations, estimated from the eight reconstructions.

The inset figure of Fig. 10 ▸(*a*) illustrates that the lengths between 1.25 and 1.35 Å show large variation compared with other distances. The inset figure of Fig. 10 ▸(*b*) shows that the bonds with angles between 115 and 125° have a similar large variation. These bond lengths and angles are in the typical range for aromatic bonding. The overlay image of Fig. 6 ▸(*c*) shows visible variation in the structure within the aromatic bonds. Despite the variance in some of the bond lengths and angles, the average is still close to the expected target value. Furthermore, the bond lengths and angles due to the heavier elements (S and Ag) within the structure are accurately reconstructed. Heavier atoms scatter more readily (Warren, 1990 ▸) and this implies their contribution to the Bragg peak intensity is higher. It then follows that their contribution to the correlation function is more apparent and, hence, has a greater influence on the recovered Bragg intensities. This is also evident in the aluminophosphate structure, where there is no observable change in the locations containing the aluminium atoms. The bond comparison plots in Fig. 11 ▸(*a*) demonstrate accurate refinement of the inorganic bonds above 1.7 Å, and higher variance in the organic bonds between 1.4 and 1.6 Å.

**Figure 12:**
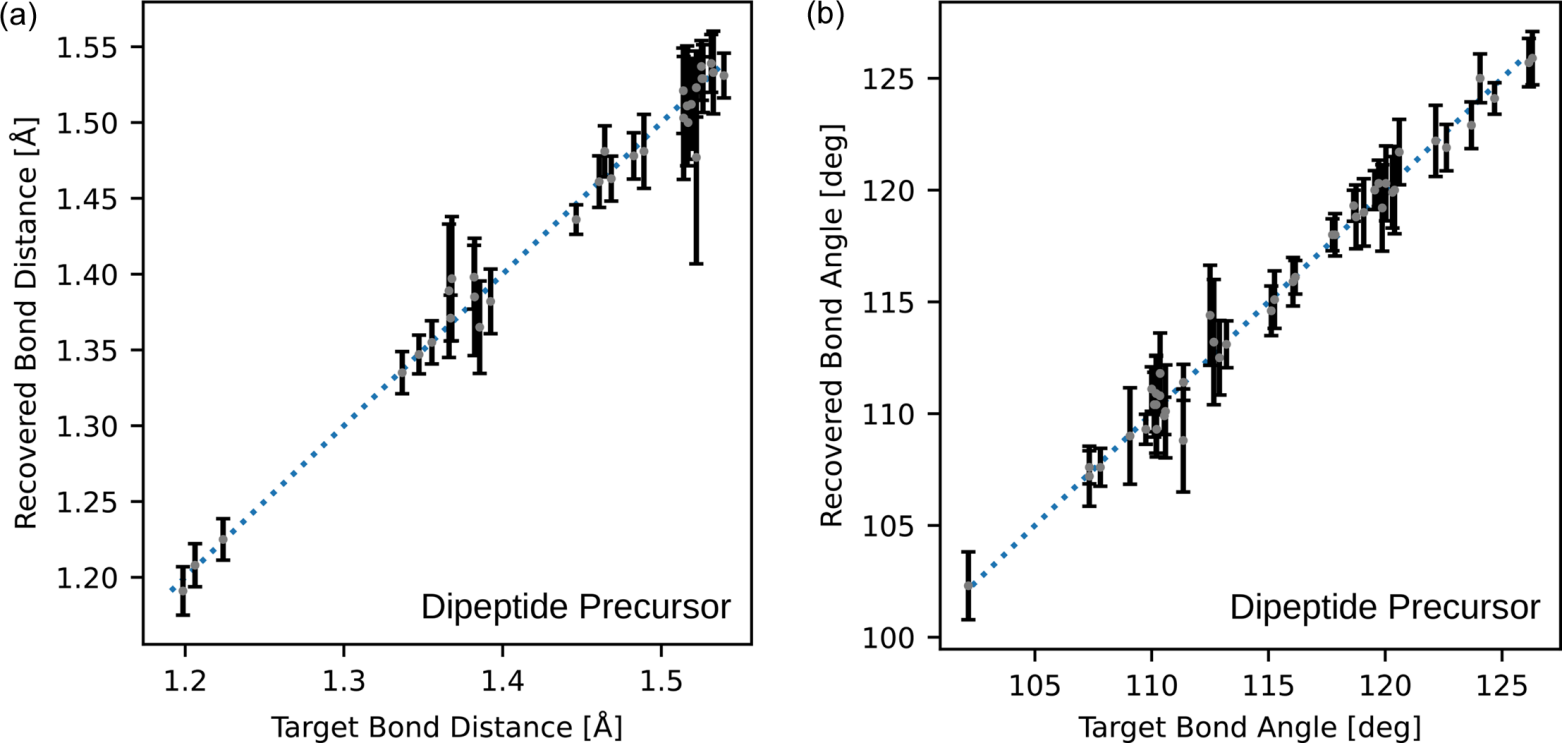
Comparison of (*a*) bond lengths and (*b*) bond angles of the target and the average structure for the dipeptide sample. Error bars are ±3 standard deviations, estimated from the eight reconstructions.

The dipeptide precursor structure has no heavier elements and the peptide bonds within this structure resemble components in proteins. The recovery of this structure is a step towards the potential application of the algorithm to macromolecular crystal structure determination. All of the elements refined equally well and refined more accurately than the lighter elements (C, N, O) of the previous two samples. This is probably due to the lack of heavy elements in the dipeptide precursor structure. This is illustrated in the bond comparison plots in Figs. 12 ▸(*a*) and 12 ▸(*b*), where similar variance is shown throughout the bond lengths and angles. The variance in the organic bond lengths and angles in the dipeptide precursor structure is smaller than the variance in the organic bond lengths and angles of the silver nitrate and aluminophosphate structures. Overall, the variance in the structures is comparable to the resolution limits of the simulated structures.

### 3.3. Radial-sampling requirements

To test the effects of radial sampling on the algorithm, we generated structure factors for the silver nitrate structure to a 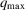 of 9 Å^−1^ or to a minimum resolution *d* of 0.7 Å. This provided 8324 scattering vectors from which we calculated six scattering correlation functions according to equation (4). These correlation functions had increasing radial sampling, where *n*_*q*_ ranged from 50 to 200 sampling points. The correlation angular-sampling parameter *n*_η_ was set to 11 520 and the harmonic order matrix was calculated to a maximum spherical harmonic of *l* = 45. The *n*_*q*_ parameter for the reciprocal-space intensity functions ranged from 50 to 200 sampling points, depending on the correlation function. The angular-sampling parameters of the intensity functions were *n*_θ_ = 360 and *n*_ϕ_ = 720.

The support peak width was 5 voxels, as in the previous structure-determination cases. The algorithm recipe consisted of 20 iterations of HIO, followed by two iterations of ER, repeated five times. The time to run this recipe increased linearly with increasing radial sampling, ranging from ∼30 min for *n*_*q*_ = 50 to 2.5 h for *n*_*q*_ = 200. The algorithm was run once for each radial-sampling parameter and the intensity was not averaged over multiple independent runs. For each iteration, we calculated the *R* factor to compare the target intensity with the recovered intensity according to equation (33). We completed *SHELXL* refinement at every iteration and calculated the mean atomic displacement according to equation (35).

**Figure 13:**
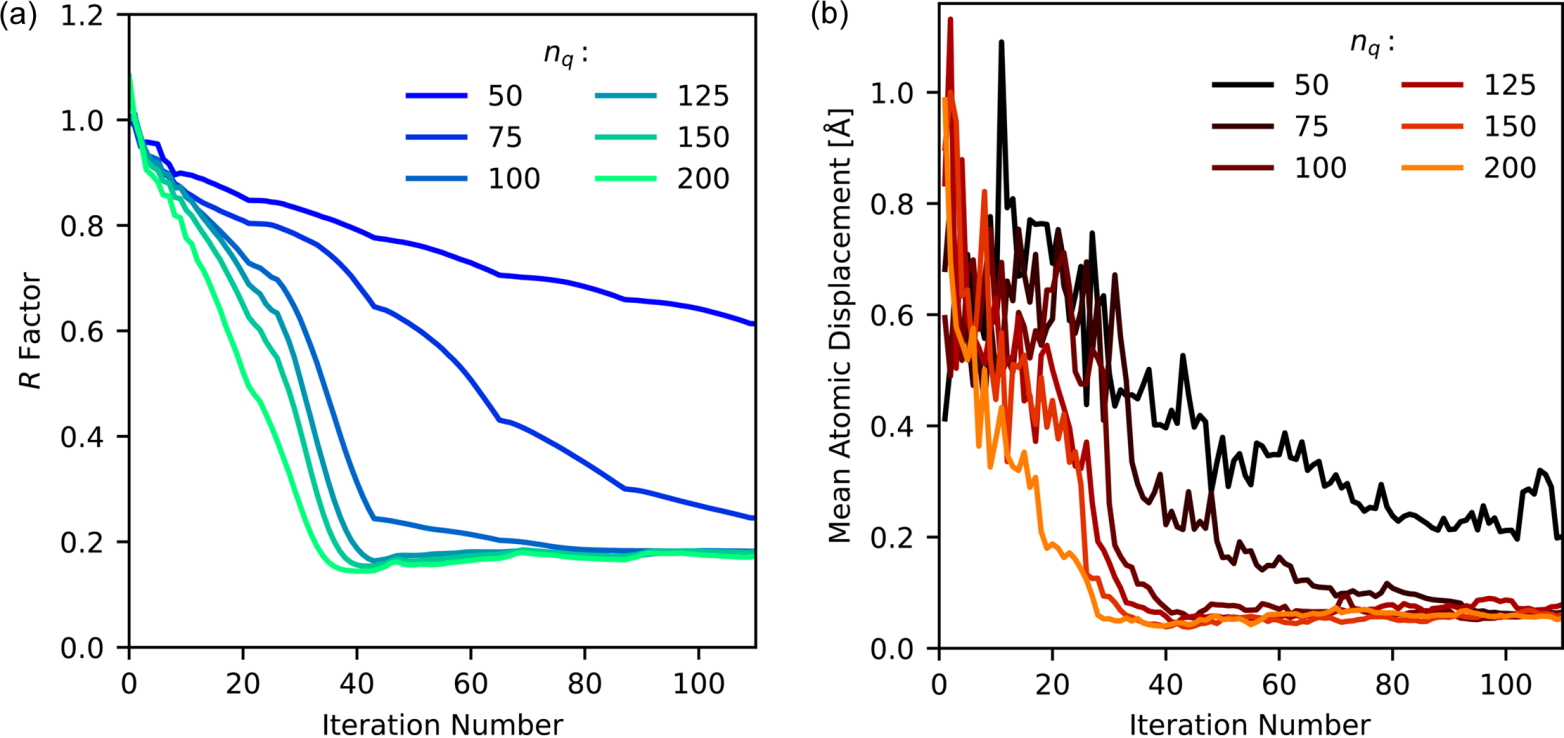
(*a*) The *R* factors and (*b*) mean atomic displacement for the silver nitrate structure as a function of iteration number for various radial-sampling conditions.

Fig. 13 ▸(*a*) illustrates that the *R* factor decreases with increasing radial sampling. When the radial sampling is increased by having more *q* points within the intensity function, fewer Bragg peaks are found within each *q* position of the intensity function. This causes less overlap between Bragg peaks in the intensity function and less overlap in correlation peaks in the correlation function. Both of these factors improve the reconstruction. As expected, increasing the radial sampling of the intensity function improves algorithm accuracy. This is supported by the plot in Fig. 13 ▸(*b*), which plots the mean atomic displacement as a function of algorithm iteration. With increasing radial sampling, the average displacement of the atoms in the structure compared with the target decreases.

In Figs. 13 ▸(*a*) and 13 ▸(*b*), there appears to be a radial sampling of *n*_*q*_ = 100 after which further increases do not improve the *R* factor or mean atomic displacement of the reconstruction. This effect is governed by the overlap of peak areas *V*_*hkl*_ in the support *M*. For example, in the reciprocal lattice of the silver nitrate crystal, the smallest *q*-axis vector magnitude is |c*| = 0.51 Å^−1^. This is the smallest distance between two adjacent Bragg peaks. With a 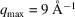 over *n*_*q*_ = 100, the size of each *q* sampling point is d*q* = 0.9 Å^−1^. Consequently, two adjacent Bragg peaks in the intensity function could be in adjacent voxels with respect to the *q* axis. The overlap is most problematic at high *q*, since there are more Bragg peaks with increasing *q*. This presents a sampling issue within the reconstruction and, hence, the *R* factor is higher for these cases. The smaller the number of sampling points in *q*, the larger the size of each sampling point over the same 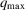. This increases the overlap between Bragg peaks in the binary support mask *M*. For radial sampling above this limit, *n*_*q*_ between 100 and 200, the *R* factor decreases sharply until iteration 40, after which the *R* factor plateaus, with marginal increase. The sharp onset of the plateau was also observed in the structure-recovery results in Fig. 5 ▸. A series of kinks in the graphs at ∼22, 44, 66, *etc*. iterations occur due to the recipe changing between the HIO and ER iterative schemes.

### 3.4. Angular-sampling requirements

To test the effect of angular sampling on the algorithm, we conducted six runs with angular sampling ranging from *n*_θ_ = 120 to *n*_θ_ = 360 sampling points. We produced a correlation function for the silver nitrate sample to a 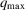 of 9 Å^−1^, or to a minimum resolution of 0.7 Å, with 11 520 sampling points for *n*_η_ and 150 sampling points for *n*_*q*_. The harmonic order matrix limit was set to 45 harmonics. The time to run the algorithm scaled quadratically with *n*_θ_, between 15 min and 1.2 h for the *n*_θ_ = 120 to *n*_θ_ = 360 runs. The quadratic scaling occurs due to increasing two axis dimensions in the intensity function, compared with increasing one axis in the radial case. When running the algorithm, we used the same recipe as in the radial-sampling case. That is, 20 iterations of HIO, followed by two iterations of ER, repeated five times. The algorithm was run once for each angular-sampling parameter, and the intensity was not averaged over multiple independent runs.

**Figure 14:**
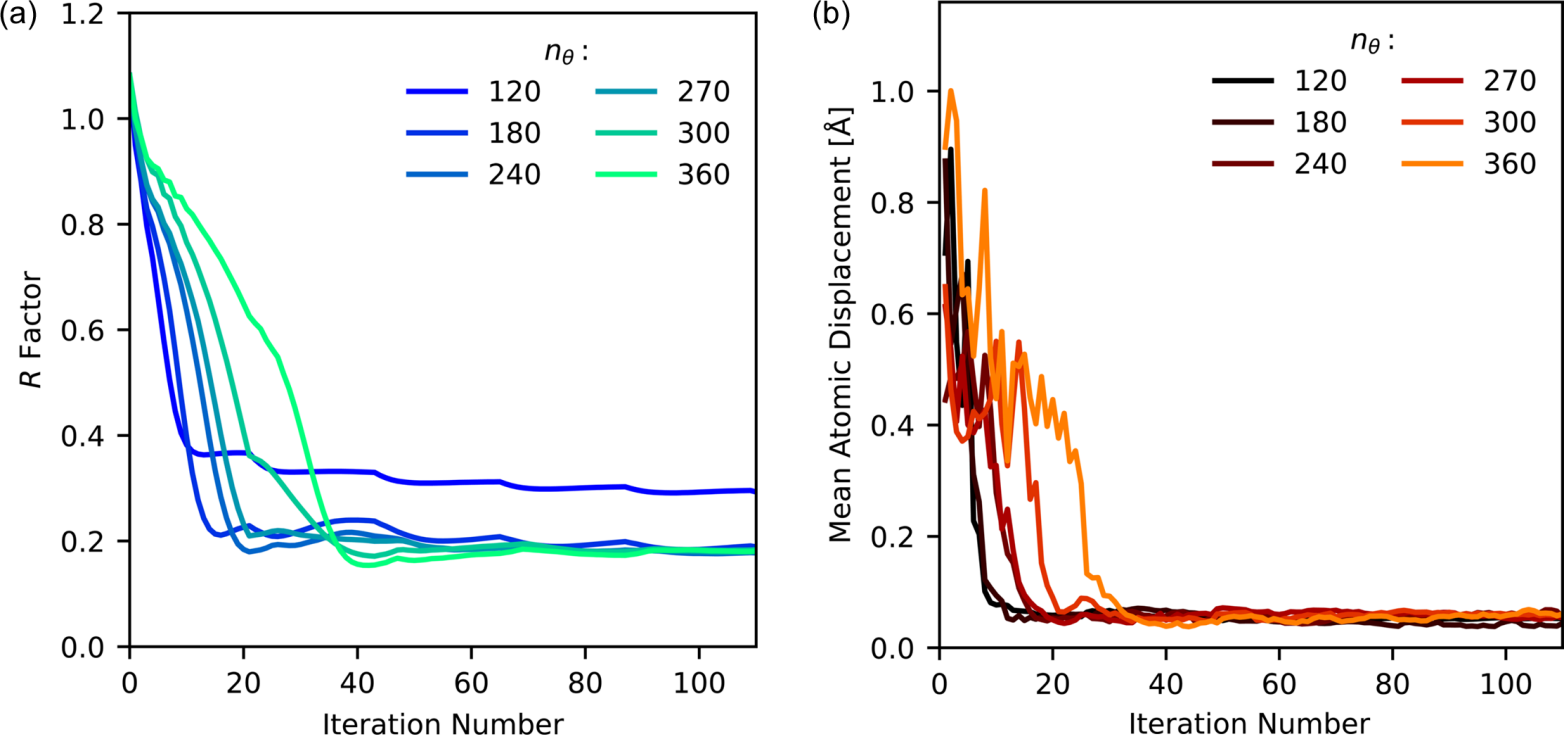
(*a*) The *R* factors and (*b*) mean atomic displacement for the silver nitrate structure as a function of iteration number for various angular-sampling conditions.

The *R* factor and mean atomic displacement as a function of iteration number are shown in Figs. 14 ▸(*a*) and 14 ▸(*b*), respectively. By decreasing the angular sampling, the onset of the plateau shifts from 40 iterations, as seen in the radial-sampling case, back to a range of 15–20 iterations. This is consistent with the structure-recovery tests in Fig. 5 ▸, where the angular sampling was higher, *n*_θ_ = 500, and the plateau begins after 60 iterations. The high angular-sampling runs have a higher *R* factor and have a slower descent before the plateau. This is also observed in the mean atomic displacement plots, where the higher angular sampling causes a slower descent into the minimum displacement value. Overall, the angular-sampling *R* factor converges at *n*_θ_ = 120 and *n*_θ_ = 180, where further increases do not change the *R* factor. This was not observed in the mean atomic displacement plot, as all the final reconstructions appear to fall within the same range. As in the radial-sampling case, kinks in the *R*-factor plot are observed where the recipe changes between HIO and ER.

### 3.5. Algorithm recipe testing

**Figure 15:**
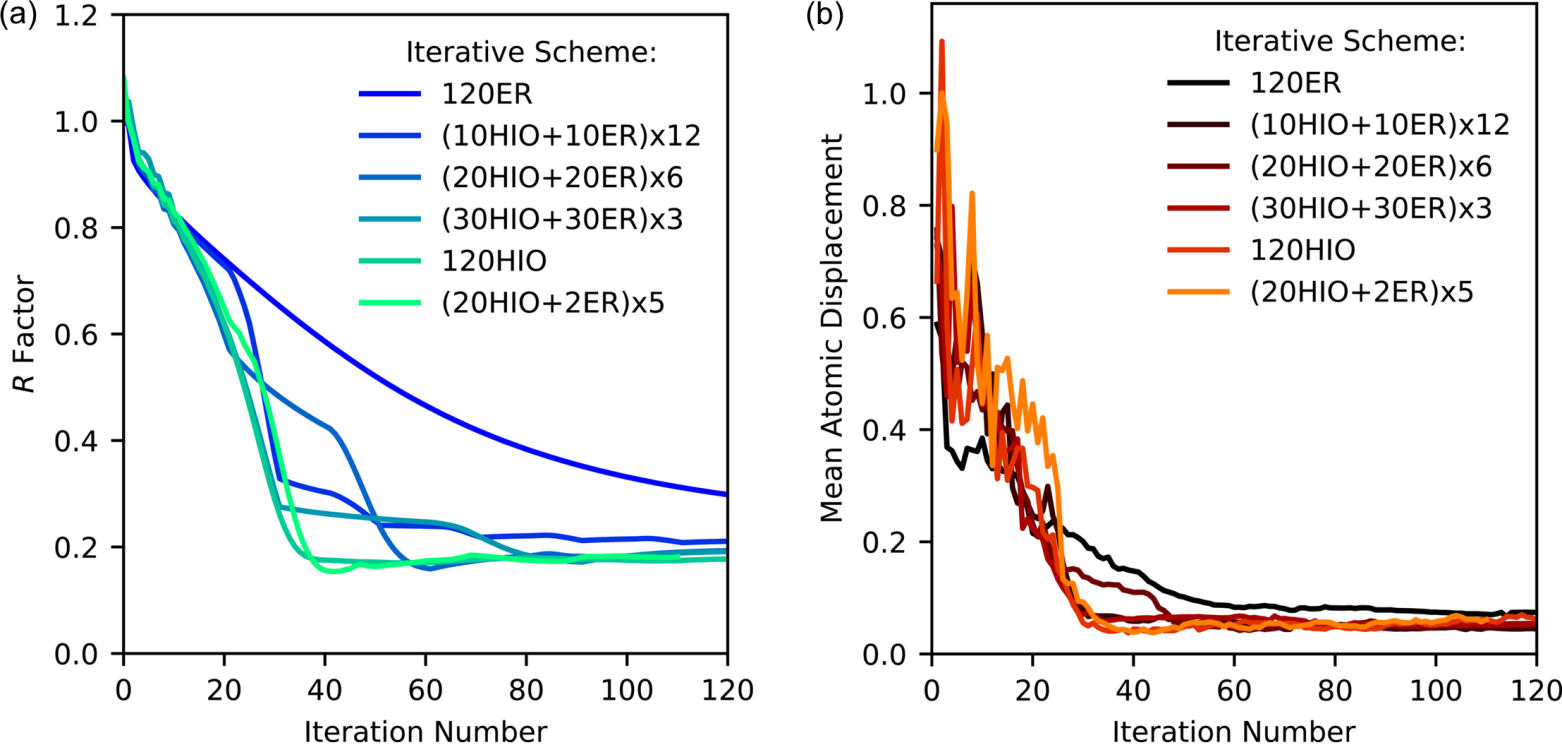
(*a*) The *R* factors and (*b*) mean atomic displacement for the silver nitrate structure as a function of iteration number for various algorithm recipes.

To test the effect of the algorithm recipe on the recovered intensities, the following recipes were tested: 120 ER, (10 HIO + 10 ER) × 12, (20 HIO + 20 ER) × 6, (30 HIO + 30 ER) × 2, (20 HIO + 2 ER) × 5, and 120 HIO. The correlation function used in this test was calculated for the silver nitrate sample to a 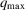 of 9 Å^−1^, or to a minimum resolution *d* of 0.7 Å, over 150 *n*_*q*_ sampling points, with 11 520 sampling points for *n*_η_ sampling. All the reconstructions used the same angular and radial sampling, *n*_θ_ = 360 and *n*_*q*_ = 150, and one reconstruction was conducted per recipe. The *R* factor and mean atomic displacement as a function of iteration number for each recipe are plotted in Figs. 15 ▸(*a*) and 15 ▸(*b*), respectively.

The *R* factor steadily decreases in the 120 ER recipe, as shown in Fig. 15 ▸(*a*). This is expected from ER, where it approaches a minimum with monotonically decreasing error (Marchesini, 2007 ▸). In Fig. 15 ▸(*b*), the mean atomic displacement of the 120 ER recipe is comparable to that of the other recipes that contain HIO. This indicates that although ER converges to the closest local minimum, it does appear to be approaching a similar solution to the recipes that include HIO. The advantage of the recipes containing HIO, however, is the speed at which the algorithm approaches the solution. The *R* factors and mean atomic displacements for the 120 HIO recipe decrease more sharply than the 120 ER recipe. Unlike the ER scheme, the HIO scheme does not necessarily monotonically decrease, due to the global minima search style of the scheme (Marchesini, 2007 ▸).

In the combination recipes, we can see a series of steps in the graph that indicate the iteration number at which the recipe changes from HIO to ER. Comparing the (30 HIO + 30 ER) × 2 and (20 HIO + 20 ER) × 6 recipes, we observe that the *R* factor plateaus at later iteration numbers, 80 and 60 iterations, respectively, compared with the minimum observed at 40 iterations in the HIO-only recipes. This indicates that the inclusion of ER in the algorithm recipe can delay the onset of the plateau in the *R* factor.

### 3.6. Protein crystal reconstruction

**Figure 16:**
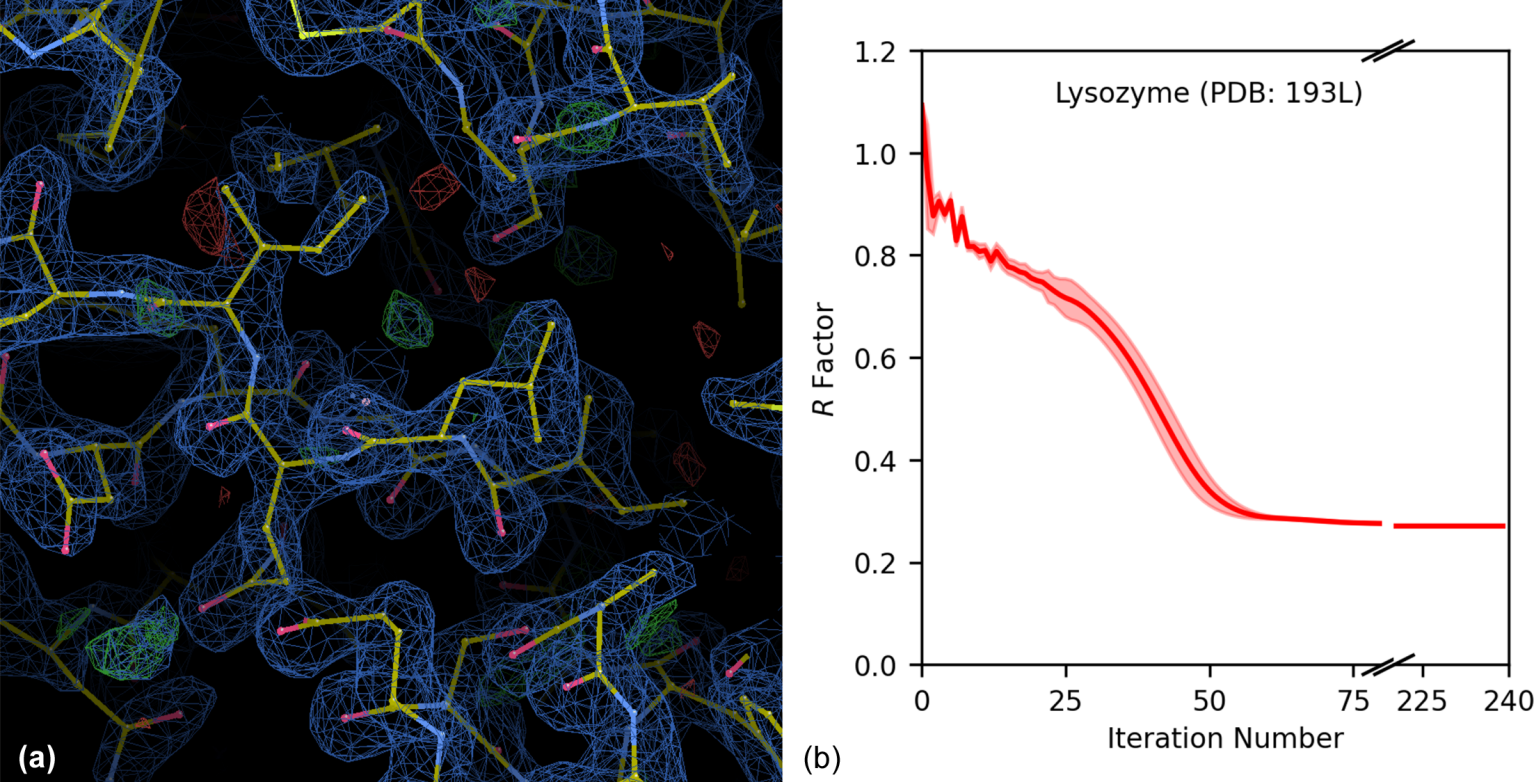
(*a*) Electron-density results of the hen egg-white lysozyme structure, from the intensities recovered with the iterative algorithm. Electron-density surfaces are plotted at 0.046e Å^−3^, with 1.5 root-mean-square deviations. The blue mesh indicates the 2*F*_o_ − *F*_c_ density, where *F*_o_ and *F*_c_ are the structure factors recovered from the algorithm after phasing and the structure factors of the model, respectively. The green and red meshes indicate the difference map *F*_o_ − *F*_c_, and show over and under represented regions of electron density, respectively. (*b*) A plot of the *R* factor versus iteration number for the lysozyme reconstruction. The solid line traces the average *R* factor over nine independent reconstructions with the same parameters but different random starts. The shaded regions above and below the average indicate ±3 standard deviations from the mean of the nine runs. The *R* factor calculated at each step decreases, indicating that the reconstructed intensities are approaching the target intensities.

Finally, we tested the algorithm’s capability in reconstructing the intensities of a hen egg-white lysozyme protein crystal structure (PDB ID 193l; Vaney *et al.*, 1996 ▸). The structure-factor intensities for the crystal were downloaded from the PDB and the 3D scattering correlation function was calculated up to *q*_max_ = 3Å^−1^, or to a minimum resolution of *d* = 2.1 Å, which included 106 124 scattering vectors. The correlation function was sampled over 23 040 *n*_η_ points and 300 *n*_*q*_ radial bins, and the radial sampling for the intensity function was *n*_θ_ = 500 and *n*_ϕ_ = 1000. The harmonic order matrix limit was set to 45 harmonics, and the support peaks had a width of 5 voxels, as in the previous examples. The algorithm recipe consisted of 240 iterations of HIO. We produced nine independent runs using these parameters. To merge the intensities, we used *AIMLESS* (Kabsch, 2010 ▸) in the *CCP4* software package (Winn *et al.*, 2011 ▸). The *R*_meas_ factor after merging was 0.1, indicating good agreement between the runs. The space group of the original crystal structure was *P*4_3_2_1_2, but, after merging, *AIMLESS* determined a *P*422 space group. We then used the merged intensities to perform basic molecular replacement with the target structure using *PHASER* (McCoy *et al.*, 2007 ▸). The structure produced from *PHASER* had an *R* factor of 0.29 and an *R*_free_ of 0.31, and the correct *P*4_3_2_1_2 symmetry was identified. A portion of the recovered protein structure and electron density is shown in Fig. 16 ▸(*a*). The average *R* factor as a function of the iteration number over the nine independent runs is plotted in Fig. 16 ▸(*b*).

## 4. Discussion

We have demonstrated that the Bragg intensities of crystal structure factors can be successfully recovered from the correlation functions of FXS analysis using an iterative algorithm. To do this, we devised a set of projection operators that modify intensity functions, which were based on the unit-cell parameters of the crystal and the correlation function that can be measured experimentally during an FXS experiment.

In our work, we used the *R* factor as a measure of algorithm accuracy, which is the typical method of assessing the refinement of a crystal structure. We quoted final *R* factors of our small-molecule reconstructions between 0.15 and 0.2. Typical *R* factors for small chemical crystals refine to less then 0.05 (IUCr, 2017 ▸), an order of magnitude smaller than we report. Despite this, the average distance between an atom in the recovered structure and the target was 0.05 Å. Furthermore, visual inspection of the final structures generated after *SHELXL* refinement confirmed that the recovery of the structure was accurate. Further improvement to the *R* factors could be achieved with finer sampling parameters for the intensity function.

The *R* factor calculated after phasing for the protein crystal reconstruction was 0.29, which is higher than was obtained for the small molecular crystals. It is also higher than the *R* factor from which the test structure was sourced (Vaney *et al.*, 1996 ▸), which was 0.23. It is typical to complete multiple iterations of refinements on a phased structure, which includes the addition of water molecules to the structure and adjusting bond parameters to better fit with the electron density. Vaney *et al.* (1996 ▸) optimized their structure with multiple iterations of refinement and the inclusion of water molecules, which we did not reproduce. The lack of water molecules in the structure could account for the regions of positive difference density in Fig. 16 ▸(*a*).

Preliminary testing regarding the effect of support peak overlap on the algorithm was also conducted. Increasing the support width for the same *n*_*q*_ and *n*_θ_ sampling parameters led to significant overlap between Bragg peaks in the support, and the algorithm did not converge. This suggests that the sampling should be selected to avoid peak overlap, but further investigation is still needed to determine if minor levels of overlap can be tolerated, and if so, to quantify how much.

Further improvements in the analysis process could be made by utilizing symmetry constraints within the crystal structure. The intensity function has a point-group symmetry related to the crystal space group (Shmueli *et al.*, 2010 ▸). This could be used as an additional constraint of the intensity function. In the algorithm we present, we have made no assumptions about the symmetry of the intensity function, including Friedel symmetry, *I*(**q**) = *I*(−**q**). Previous calculations of harmonic order matrices often excluded odd-order harmonics (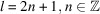), under the assumption that Friedel symmetry is preserved (Martin, 2017 ▸; Donatelli *et al.*, 2017 ▸). The algorithm we have developed currently includes odd-order harmonics in all calculations. Excluding odd-order harmonics could potentially improve algorithm accuracy and improve the speed of calculation, halving the number of harmonics that are calculated at each algorithm step.

All our algorithm testing was performed on correlations calculated from 3D scattering vectors, which, in principle, are equivalent to a converged correlation function from 2D scattering vectors. Peaks in 2D patterns are partial reflections and the convergence of the 2D correlation function performs a type of Monte Carlo integration on peaks in the correlation function. The convergence of the Monte Carlo integration of indexed reflections is well established for serial crystallography (Kirian *et al.*, 2011 ▸), but not for correlation functions. Although a full study of this convergence is beyond the scope of this work, we briefly summarize here some preliminary tests. We have performed simulations with pattern_sim from the *CrystFEL* package (White *et al.*, 2012 ▸) that compare the correlation function from simulated 2D diffraction patterns of lysozyme crystals with the correlation function from the 3D Bragg vectors of the lysozyme. The convergence between the 2D and 3D correlation functions depends on many factors, such as the sampling of the functions in terms of *n*_*q*_, *n*_ψ_, 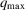, the number of patterns used to calculate the 2D correlation function, the unit-cell dimensions and the size of the crystal. After ∼10^5^ simulated diffraction patterns, the location and relative intensity of peaks within the 2D correlation function had a clear resemblance to peaks within the 3D correlation function. However, peaks in the 2D correlation function tend to spread due to the width of the Bragg peaks in the diffraction patterns. This essentially creates a blurred appearance for the 3D correlation function. The effect of this blurring on the algorithm recovery is unknown and requires further investigation. There are some other numerical differences that can arise between the two cases. The 2D correlation function requires a factor of 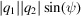, which, as previously stated, accounts for the curvature of the Ewald sphere and to maintain even sampling of ψ. The size of the Bragg peaks in the diffraction patterns also affects the convergence of the functions. With larger Bragg peaks, each correlation peak spreads depending on the spread of the Bragg peak in reciprocal space. This can be accounted for by blurring the 3D correlation function, or integrating peaks in the 2D correlation function to the positions in the 3D correlation function. With regards to experimentally calculating the correlation function from crystals, convergence of the 2D correlation function to the 3D correlation function can depend on various factors such as the signal-to-noise ratio, number of crystals per pattern, the distribution of the crystal size and preferred orientation. The effect of these factors on the ability to obtain a correct and ideal 3D correlation function requires further investigation. Further simulation work on 2D crystal correlations would help illuminate the convergence issues and identify how much data are required in an experiment.

## 5. Conclusions

We have demonstrated the extraction of crystal structure-factor amplitudes from FXS correlation functions through the use of an iterative algorithm. We constructed a set of projection operators for an iterative algorithm that recovers the 3D reciprocal-space intensity of a crystal from a random starting point. The algorithm was successfully tested on three small chemical crystal structures and a protein crystal structure. It was shown that the sampling should be sufficient to avoid peak overlaps to improve performance. This approach could be further developed in the future to facilitate the extraction of structure factors from spotty powder patterns collected from sub-micrometre chemical crystals, and could open the door to novel structural-determination techniques through the use of fluctuation-scattering analysis.

## Figures and Tables

**Figure 8:**
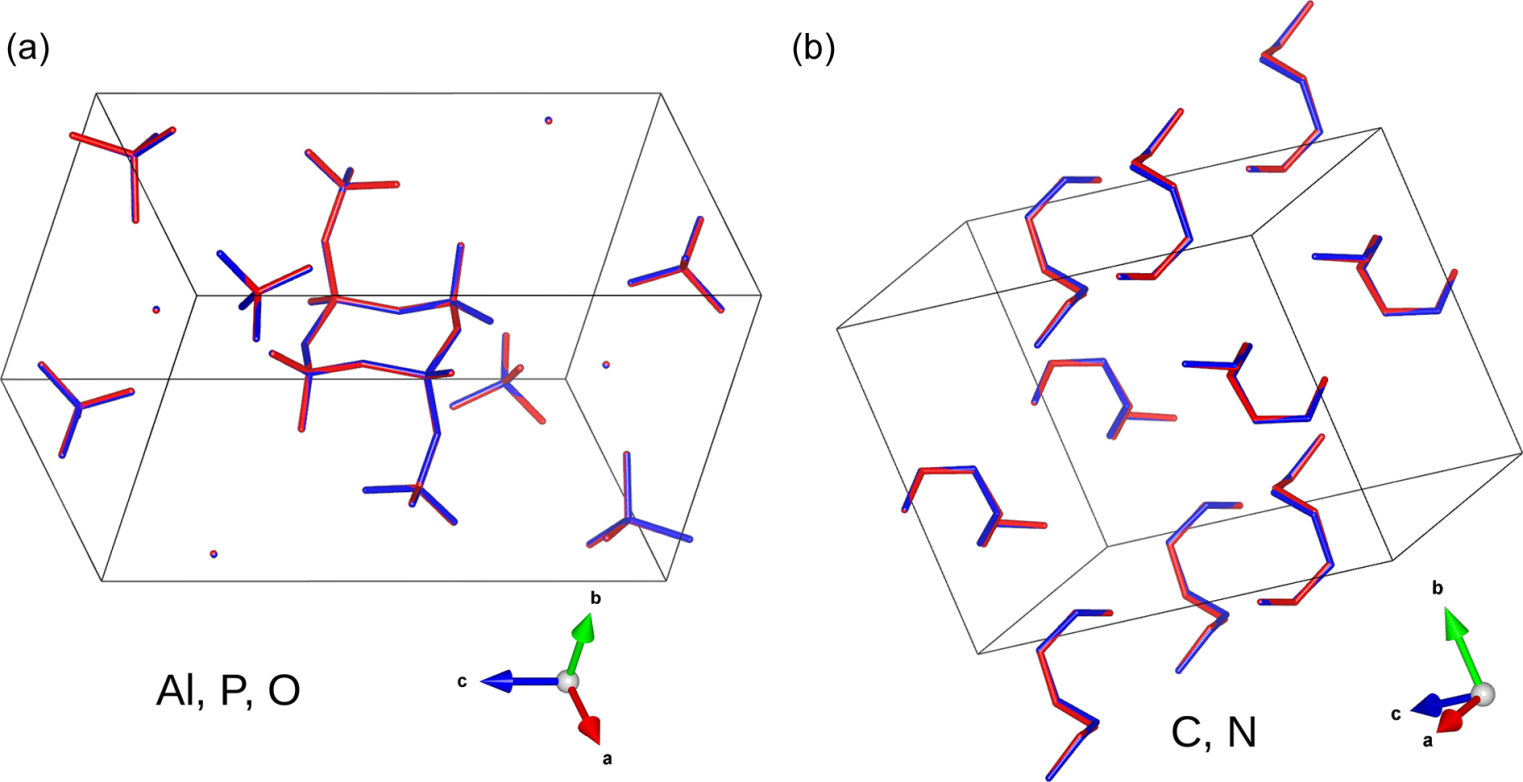
Overlays of the blue target and red recovered structures for aluminophosphate. (*a*) Overlays only including Al, P and O atoms in the structure. (*b*) Overlays only including C and N atoms in the structure.

**Figure 9:**
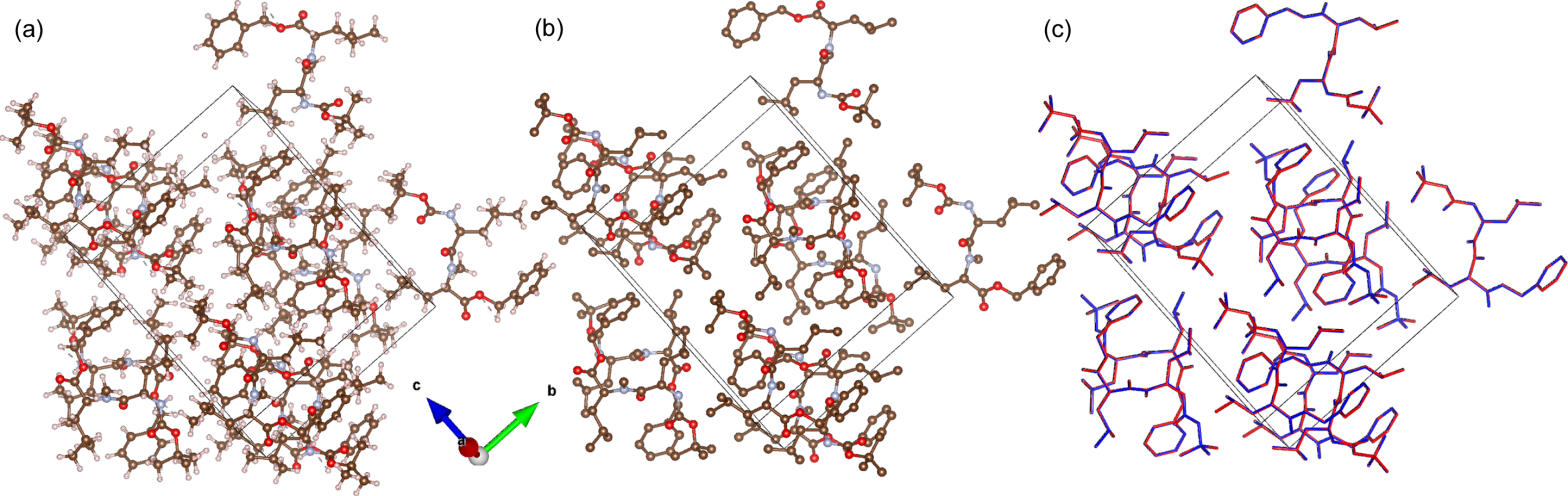
(*a*) The target and (*b*) the recovered structures for the dipeptide precursor sample. (*c*) The overlay of the blue target and red recovered structures.

**Table 1:** Crystal structure data used for testing the algorithm Quantities *a*, *b*, *c* refer to the magnitudes of the unit-cell vectors. Quantities α, β, γ refer to the angles between the lattice vectors.

Structure	Silver nitrate/ligand	Aluminophosphate	Dipeptide precursor
Formula	C_10_H_14_AgN_2_O_5_S	(C_5_H_16_N_2_)[AlP_2_O_8_]	C_25_H_40_N_2_O_5_
Lattice/Sym.	Triclinic (*P* 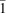 )	Monoclinic (*P*2_1_/*n*)	Orthorhombic (*P*2_1_2_1_2_1_)
*a* (Å)	5.187 (2)	7.8783 (2)	9.9400 (12)
*b* (Å)	10.722 (3)	10.46890 (10)	14.9395 (18)
*c* (Å)	12.636 (4)	16.0680 (4)	17.876 (2)
α (°)	82.315 (4)	90	90
β (°)	78.712 (4)	95.1470 (10)	90
γ (°)	79.952 (4)	90	90
Reference	Hanton & Lee (2000 ▸)	Phan Thanh *et al.* (2000 ▸)	Liao *et al.* (2007 ▸)
